# Restoring STING expression in soft tissue sarcoma increases activation and function of tumor-infiltrating lymphocytes

**DOI:** 10.1007/s00262-026-04455-3

**Published:** 2026-06-21

**Authors:** Stine Høvring Godsk, Mireia Cruz De los Santos, Tobias Wang Bjerg, Thomas Gravgaard Andersen, Felix Haglund de Flon, Maria Elisabeth Harder, Anders Etzerodt, Ninna Aggerholm-Pedersen, Andreas Lundqvist, Martin Roelsgaard Jakobsen

**Affiliations:** 1 Department of Biomedicine, Aarhus University, 8000 Aarhus C, Denmark; 2 Department of Oncology-Pathology, Karolinska Institutet, 17177 Stockholm, Sweden; 3 Department of Oncology, Aarhus University Hospital, 8200 Aarhus N, Denmark

**Keywords:** Sarcoma, STING, TILs, Immunotherapy

## Abstract

Sarcomas are rare, highly heterogeneous malignancies, with soft tissue sarcomas (STS) comprising nearly 80 histological subtypes. Localized STS is generally treated with surgery and radiotherapy, whereas metastatic disease relies largely on chemotherapy, which offers limited benefit. Although inflammation influences tumor development, treatment response, and prognosis, immune checkpoint inhibitors have shown minimal efficacy in sarcoma. The cyclic GMP–AMP synthase (cGAS)–stimulator of interferon genes (STING) pathway is linked to mutational burden, genomic instability, immune cell infiltration, and therapeutic response across multiple cancer types. However, its role in sarcoma remains unclear. In a newly established cohort of STS patients, we found that STING expression in primary tumors correlates with immune cell infiltration. Using a lipid nanoparticle (LNP)–mediated delivery of CRISPR activation mRNA components, we restored STING expression in STS cells, thereby reactivating cGAS–STING signaling. This promoted T‑cell recognition and tumor cell killing in both 2D and 3D patient-derived models and enhanced responses to anti-PD-1 treatment. Together, our results show that controlled epigenetic activation of STING in STS enhances immune infiltration and tumor cell killing. Targeting STING through CRISPR activation may therefore represent a promising therapeutic strategy for STS.

**Supplementary Information:**

The online version contains supplementary material available at https://doi.org/10.1007/s00262-026-04455-3.

## Introduction

Sarcomas are rare malignancies, representing only 1% of all cancers but 12–15% of pediatric tumors. They include bone sarcomas and soft tissue sarcomas (STS) [1], the latter comprising approximately 80 histological subtypes that arise across nearly all anatomical sites [1, 2]. While localized STS has a five-year survival rate of 70–80%, metastatic disease remains associated with survival rates below 20% [1, 3]. Despite the impact of immune checkpoint inhibitors (ICI) in other cancers, their efficacy in metastatic STS has been limited, with clinical benefit largely restricted to selected histologies, such as undifferentiated pleomorphic sarcoma (UPS), angiosarcoma, and alveolar soft part sarcoma [4–6]. Current treatments consist primarily of surgery with radiotherapy for localized disease and doxorubicin-based systemic therapy for metastatic disease. However, response rates remain modest (12–24%), and long-term survival is not significantly improved [3, 7, 8]. The low mutational burden of STS [4, 9], compared with highly mutated tumors such as non-small cell lung cancer [10] and melanoma [11], likely contributes to poor ICI responsiveness. However, high immune infiltration remains associated with improved survival [4, 10, 12]. Increasing evidence links mutational burden, genomic instability, and immune infiltration to activation of the stimulator of interferon genes (STING) pathway [13], a key innate immune sensor that drives antitumor responses within the tumor microenvironment (TME) [14]. STING is activated by cytosolic DNA arising from genomic instability [15] or from DNA-damaging therapies [16, 17], initiating cGAS-cGAMP signaling that induces type I interferons [18] and downstream interferon-stimulated genes (ISGs) [19]. STING agonists induce tumor regression in vivo [20, 21] and have been explored in combination with immunotherapy [20–24]. Conversely, epigenetic STING downregulation has been linked to ICI resistance and reduced immune infiltration [17, 25].

Despite these advances, the role of STING in STS remains poorly defined. In this study, we examined STING regulation and its relationship to T-cell-mediated antitumor activity. We found that STING expression correlates with immune infiltration in a large STS cohort and identified STING suppression as a potential immune-evasion mechanism. Restoration of STING via CRISPR activation (CRISPRa) rescued pathway functionality and cytokine production. Furthermore, lipid nanoparticle (LNP)-mediated delivery of STING CRISPRa in sarcoma patient-derived ex vivo models restored tumor recognition and killing by autologous tumor-infiltrating lymphocytes (TILs) and improved responses to ICI.

## Materials and methods

### RNA sequencing analysis in sarcoma patient cohort and TCGA database

Patients referred to Aarhus Sarcoma Center between 2014 and 2018 were included in this study. Patient tissue used for RNA sequencing was stored at the National Cancer Biobank, Aarhus, Denmark, until analysis. Paired-end RNA sequencing was performed on 136 patient samples by MOMA NGS Core Center, Aarhus, on an Illumina NovaSeq 6000 instrument. Patients diagnosed with aggressive soft tissue sarcoma (*N* = 120) were included in the analysis. Data analysis was performed in R version 4.4.1. TCGA pan-cancer and TCGA Sarcoma datasets were analyzed in RStudio (version 4.4.1). Clinical and gene expression data were obtained from Xena UCSC. Immune cell infiltrates were predicted by calculating scores based on the genes provided in Supplementary Table 1.

### Cell culture of commercial cell lines

HT-1080, SW872, SW982, and SK-LMS1 were purchased from ATCC. Cells were cultured in DMEM (Sigma-Aldrich, Cat#: D6429), supplemented with 10% fetal bovine serum (Sigma-Aldrich, Cat#: F9665), 1% L-glutamine (Thermo Fisher Scientific, Cat#: 25030024), 1% penicillin–streptomycin (Thermo Fisher Scientific, Cat#: 15140122), and 1% non-essential amino acids (Sigma-Aldrich, Cat#: M7145). The cells were grown at 37 °C and 5% CO_2_.

### Transfection

Cells were transfected 24 h after seeding in Nunc-6 or Nunc-24 plates using lipofectamine 2000 (Invitrogen, Cat# 11668019) according to the manufacturer’s instructions. Double-stranded herring testis DNA (Sigma-Aldrich, Cat# D6898-250) was used at 1 or 2 µg/mL, and polyinosinic–polycytidylic acid [poly(I:C)-LMW] (InvivoGen, Cat# tlrl-picw) at 40 ng/mL. Transfections were performed for 6 h for experiments involving cell lysates (western blotting) or co-culture, and for 24 h when supernatants were collected for protein analysis.

### Western blot

Cells were lysed in RIPA buffer (Thermo Scientific, Cat# 89900) supplemented with protease inhibitor (Millipore Sigma, Cat# 11873580001), phosphatase inhibitor (Thermo Fisher Scientific, Cat# A32961), and 10 mM NaF (VWR, Cat# J60251.AE). Lysates were mixed 1:1 with Laemmli buffer (Sigma-Aldrich, Cat# 38733), heated at 95 °C for 5 min, separated on 10% or 4–20% Criterion™ TGX™ precast gels (Bio-Rad, Cat# 567-1035 or 567-1095) using MOPS running buffer (Thermo Fisher Scientific, Cat# NP0001), and transferred to Turbo Transfer Midi PVDF membranes (Bio-Rad, Cat# 170-4157).

Membranes were blocked with 5% skimmed milk (Sigma-Aldrich, Cat# 70166), incubated with primary antibodies overnight at 4 °C, followed by HRP-conjugated secondary antibodies for 1 h at room temperature. Proteins were detected using Clarity Western ECL Substrate (Bio-Rad, Cat# 1705060) or SuperSignal West Femto Maximum Sensitivity Substrate (Thermo Fisher Scientific, Cat# 34,095) and imaged on an Azure Biosystems 300 system.

Primary antibodies: Cell Signaling Technology: anti-TBK1 Cat#: 3313S (84 kDa), anti-STING Cat#: 13647S (33–42 kDa), anti-cGAS Cat#: 15102S (62 kDa), anti-IRF3 Cat#: 4302S (55 kDa), anti-pTBK1 Cat#: 5483S (84 kDa), and anti-pSTING Cat#: 19781S (33–42 kDa). Sigma Life Sciences: anti-Vinculin Cat#: V9131 (116 kDa). Abcam: anti-pIRF3 Cat#: 76,493 (55 kDa). Secondary antibodies: Jackson ImmunoResearch: Peroxidase-AffiniPure F(ab′)2 Fragment Donkey Anti-Rabbit IgG Cat#: 711-036-152, Peroxidase-AffiniPure F(ab′)2 Fragment Donkey Anti-Mouse IgG Cat#: 715–036-150.

### RNA extraction and cDNA synthesis

RNA was extracted using NucleoSpin RNA kit (Macherey–Nagel, Cat#: 740955) or PureLink RNA Mini Kit (Invitrogen, Cat#: 12183018A). 250 ng to 1 μg of RNA was used for cDNA synthesis using the iScript cDNA synthesis kit (Bio-Rad Cat#: 1708891BUN) or cDNA was synthesized using SuperScript IV Reverse Transcriptase (Invitrogen, Cat#: 18090050), dNTP (Thermo scientific, Cat#: R0192), RNaseOUT RNase Inhibitor (Invitrogen, Cat#: 100000840), and Oligo d(T)20 primer (Invitrogen, Cat#: 58862) according to manufacturers’ instructions.

### RT-qPCR

mRNA expression was quantified using primers designed with Primer3 or obtained from ORIGENE and synthesized by Eurofins or Integrated DNA Technologies (Supplementary Table 3). qPCR was performed using KAPA SYBR Fast Master Mix (Sigma-Aldrich, Cat# KK4611) or PowerUP SYBR Green Master Mix (Applied Biosystems, Cat# A25742) with 200–400 nM primers and 10–20 ng cDNA. Amplification was carried out on a LightCycler 480 or QuantStudio 7 Flex system. Ct values were used to calculate expression levels (2^ − Ct), normalized to a reference gene, with 2–3 technical replicates averaged per sample.

### Functional type I interferon cell reporter assay

Type I IFN in supernatants from cell culture was measured using human HEK-Blue IFN-α/β reporter cells (InvivoGen). HEK-Blue IFN-α/β reporter cells were seeded in 150 μL of culture media one day before the assay started. Fifty microliters of supernatants or a standard curve dilution of IFN-α starting at 1000U/mL (PBL Assay Science, Cat#: 11100-1) were added to the HEK-Blue IFN-α/β reporter cells and left overnight. Twenty microliters of supernatant from HEK-Blue IFN-α/β reporter cells were then mixed with 180μL QUANTI-blue (InvivoGen, Cat#: rep-qb2), and absorbance was measured at 620 nm to determine U/mL.

### ELISA

CCL5, CXCL10, and IL-6 levels in cell culture supernatants were measured using Human DuoSet ELISA kits (R&D Systems, Cat# DY278, DY266, and DY206) according to the manufacturer’s instructions. Absorbance was read at 450 and 570 nm, with 570 nm values subtracted from 450 nm readings. Concentrations were calculated using a 4-parameter logistic (4PL) regression (*X* = log concentration) in Gen5 software v3.13 (BioTek).

IFNγ in co-culture supernatants was measured using a Human IFN-γ ELISA kit (MABTECH, Cat# 3420-1H-20) with minor modifications: The standard curve ranged from 10,000 to 1.24 pg/mL. Absorbance was measured at 450 nm using a VersaMax microplate reader, and concentrations were calculated by 4PL regression (*X* = log concentration) in GraphPad Prism 10.

### CRISPR-mediated transcriptional activation of *STING*

STING transcription was activated using chemically modified single-guide RNAs (sgRNAs) and a nuclease-deficient Cas9 fused to p65, Rta, and VP64 (dCas9-VPR). sgRNAs targeting the STING promoter were designed using CRISPOR and CRISPick and synthesized by Synthego with 2′-O-methyl-3′ phosphorothioate modifications at both termini. Five sgRNAs were tested individually or in combination. dCas9-VPR mRNA was in vitro transcribed from a plasmid provided by Rasmus O. Bak (Aarhus University). STING transcriptional activation by electroporation was performed by delivering 1 µg of each sgRNA and 0.5 µg dCas9-VPR mRNA into 500,000 cells using a 4D-Nucleofector (Lonza) with Nucleovette strips and program CM138-P3. An electroporated sample without sgRNA or dCas9-VPR mRNA served as control. sgRNA sequences are provided in Supplementary Table 4. For LNP delivery, CRISPRa component doses were calculated relative to 200,000 cells. LNPs were prepared using the NanoAssemblr® Ignite platform (Cytiva) using a lipid formulation as described [26] in accordance with manufacturer’s instructions. Briefly, the combined RNA (sgRNA + dCas9-VPR mRNA, ratio 2:1 w/w) in 100 μM sodium acetate buffer (pH 4.5) was mixed with the lipid phase at a 4:1 (aqueous:lipid) flow rate ratio and a total flow rate of 5 mL/min. After formulation, LNPs were dialyzed twice overnight against 1 × PBS in 14 kDa MWCO tubing. Following, LNPs were sterile filtered using cellulose acetate 0.2 μm membrane filter (Avantec®) and LNP-lipid concentration was analyzed using high-performance liquid chromatography on a Dionex Ultimate 3000 HPLC system (Thermo Fisher Scientific) equipped with a Nucleosil 100-3C18 column. RNA concentration was determined using the modified Quant-iT RiboGreen RNA (Thermo Scientific #R11490) protocol proposed by Precision NanoSystems. To ensure long-term stability of the RNA, LNPs were stored in − 70 °C, using 5% sucrose (Merck) as a cryoprotectant [27]. The size of LNPs was determined to be 110.6 nm, polydispersity 0.084, and encapsulation efficiency 94%. Cells treated with sucrose alone or LNPs containing only dCas9-VPR mRNA were used as controls.

### Flow cytometry

Cells were assessed for viability using 1:1000 Dead Cell Marker Aqua (Invitrogen, Cat#: L34966A), 1:500 LIVE/DEAD™ Fixable Blue (Invitrogen, Cat#: L34962), or 1:1000 LIVE/DEAD™ Fixable Near IR (780) (Invitrogen, Cat#: L34994) in PBS for 15 min, RT. Cell surface markers were stained for 30 min at 4ºC in PBS 5% FBS + 2 mM EDTA + 0.09% Na-azide (Invitrogen, Cat#: 15575-038). For intracellular and intranuclear markers, cells were stained using the Foxp3/Transcription-Factor Set (eBioscience, Cat#: 00-5523-00) following the manufacturer’s instructions. Intracellular markers were stained 60 min at RT or overnight, and samples acquired with Novocyte Quanteon (ACEA Biosciences), CytoFLEX S (Beckman Coulter), or ID7000 Spectral Cell Analyzer (Sony Biotechnologies). FlowJo (Tree Star) was used for analysis. Antibodies are listed in Supplementary Table 5, and representative gating strategies are shown in Supplementary Figure.

### Patient-derived sarcoma cell line establishment

Fresh surgical resections of sarcoma samples obtained from the Karolinska Hospital were processed by chemical and mechanical dissociation using the Tumor Dissociation Kit (Miltenyi, Cat 130-095-929). Selection of sarcoma tumor cells was performed using Tumor Cell Isolation Kit (Miltenyi) from the resultant single-cell suspension following the manufacturer’s instructions. Cells were cultured in DMEM GlutaMAX (Gibco, 61965-026), 1% penicillin/streptomycin (Gibco, 15140-122), 10% heat-inactivated fetal bovine serum (FBS) (Gibco, A5256701), 1 × sodium pyruvate (Gibco, 11360-039), and Primocin (InvivoGen, NC9392943).

### Expansion of tumor-infiltrating lymphocytes

Single-cell suspensions from digested sarcoma resections were cultured in (47% X-VIVO 20 (Lonza, 04-448Q), 47% AIM V medium (Gibco, 12055-091), 5% hAB serum (Karolinska Hospital) supplemented with 3000 IU/mL IL-15 (Thermo Fisher Scientific, AF-200-15-100UG)). Following one week of incubation, PBMCs from at least three donors were pooled and irradiated (40 Gy). Feeder cells were added in a 200:1 ratio to TILs and incubated in Aim V supplemented with 2% hAB serum containing 500 IU/mL IL-15 and 30 ng/ml Ultra-LEAF Purified anti-human CD3 (BioLegend, 317302). After 5, 9, and 12 days, media was refreshed and supplemented with 500 IU/mL IL-15. TILs were analyzed for purity after 10 days of culture and expanded until day 20.

### Tumor and tumor-infiltrating lymphocyte co-culture for IFNγ release assay

Treated patient-derived tumor cells were seeded at a density of 40.000 cells per well. HLA class I was blocked by pre-incubating tumor cells with 10 μg/ml Ultra-LEAF anti-HLA-A,B,C antibody (clone W6/32, BioLegend, 311427) for 1 h, where indicated. Tumor cells and TILs were co-cultured for 24 h at a 1:5 target-to-effector ratio in AIM V medium (Gibco), supplemented with 2% human AB serum (Karolinska Hospital). Thereafter, supernatants were obtained for IFNγ ELISA as mentioned above.

### Sarcoma patient-derived spheroid formation and co-culture

In Nunclon Sphera 96-well U-bottom plates (Thermo Fisher Scientific, 174929), 5000 patient-derived sarcoma cells/well were seeded in DMEM/F-12 (Gibco, 31330-038),1% penicillin/streptomycin, 1 × GlutaMAX (Gibco, 35050-061), 20% of heat-inactivated FBS, and 2% Geltrex (Gibco, A14132-02). After three days, spheroids were formed and treated with corresponding concentrations of LNPs (dCas9 LNP, STING LNP, or vehicle (sucrose)) for two additional days. If co-culture with TILs was performed, spheroids were washed 3 × with PBS before co-culture with patient-matched TILs in a 5:1 (Effector to Target) ratio.

### Real-time TILs-mediated caspase 3/7 killing assay and tumor-TIL growth control

To assess TIL-mediated killing upon co-culture with spheroids, CellEvent Caspase 3/7 Green (Thermo Fisher Scientific, C10723) was added to a final concentration of 1:5000. To assess TIL localization in spheroids, TILs were stained with 1:1000 CellTrace Far Red (Invitrogen, C34572) prior to co-culture. For PD-1 blockade, TILs were pre-incubated with Nivolumab (25 µg/ml) 1 h prior to co-culture. Nivolumab was also added at the beginning of the co-culture. Co-cultures were imaged for 48 h using IncuCyte S3 ESSEN or SX5 (Bioscience), in a humidified incubator (37 °C and 5% CO_2_). Brightfield, phase contrast, green fluorescence, and far-red fluorescence images under a 4 × objective were acquired. Analysis was performed in IncuCyte 2022B/2024 Rev 2 GUI software. Briefly, the software was trained to define spheroids from at least 30 randomly selected spheroids at different time points. The green mean intensity value, green calibrated unit (GCU), or near-infrared calibrated unit (NIRCU) within the largest spheroid were taken for each spheroid and time point.

### Single-cell suspension from spheroids

To assess infiltration of autologous TILs within the spheroid after 48 h of co-culture, spheroids were washed with PBS to eliminate non-infiltrated TILs. Six spheroids per condition were pooled and dissociated with TrypLE Express (Gibco, Cat#: 12604-013) for 5 min at 37 °C. Obtained single-cell suspensions were processed for flow cytometry (FC). This dissociation protocol was also employed to study STING expression by FC on mono-cultured spheroids.

### 3D Confocal imaging for TIL infiltration

After 48 h of spheroid-TIL co-culture, spheroids were washed 3 × with PBS to remove non-infiltrating TILs. Fixation, staining, imaging, and analysis were performed as previously described [28, 29].

### Statistics

Statistical analyses were performed using GraphPad Prism 10 and R (v4.4.1), as specified in the figure legends. Two-group comparisons were analyzed by Student’s t-test, and multiple-group comparisons by two-way ANOVA with appropriate multiple-comparison correction. Killing assays were analyzed by two-way ANOVA assessing time, treatment, and their interaction, with the interaction term reported. Spearman correlation was used for association analyses. Survival differences (overall and progression-free survival, Aarhus cohort) were evaluated by log-rank test. Statistical significance was set at α = 0.05 unless otherwise stated.

## Results

### STS exhibit heterogeneous and generally low immune infiltration

**Fig. 1:**
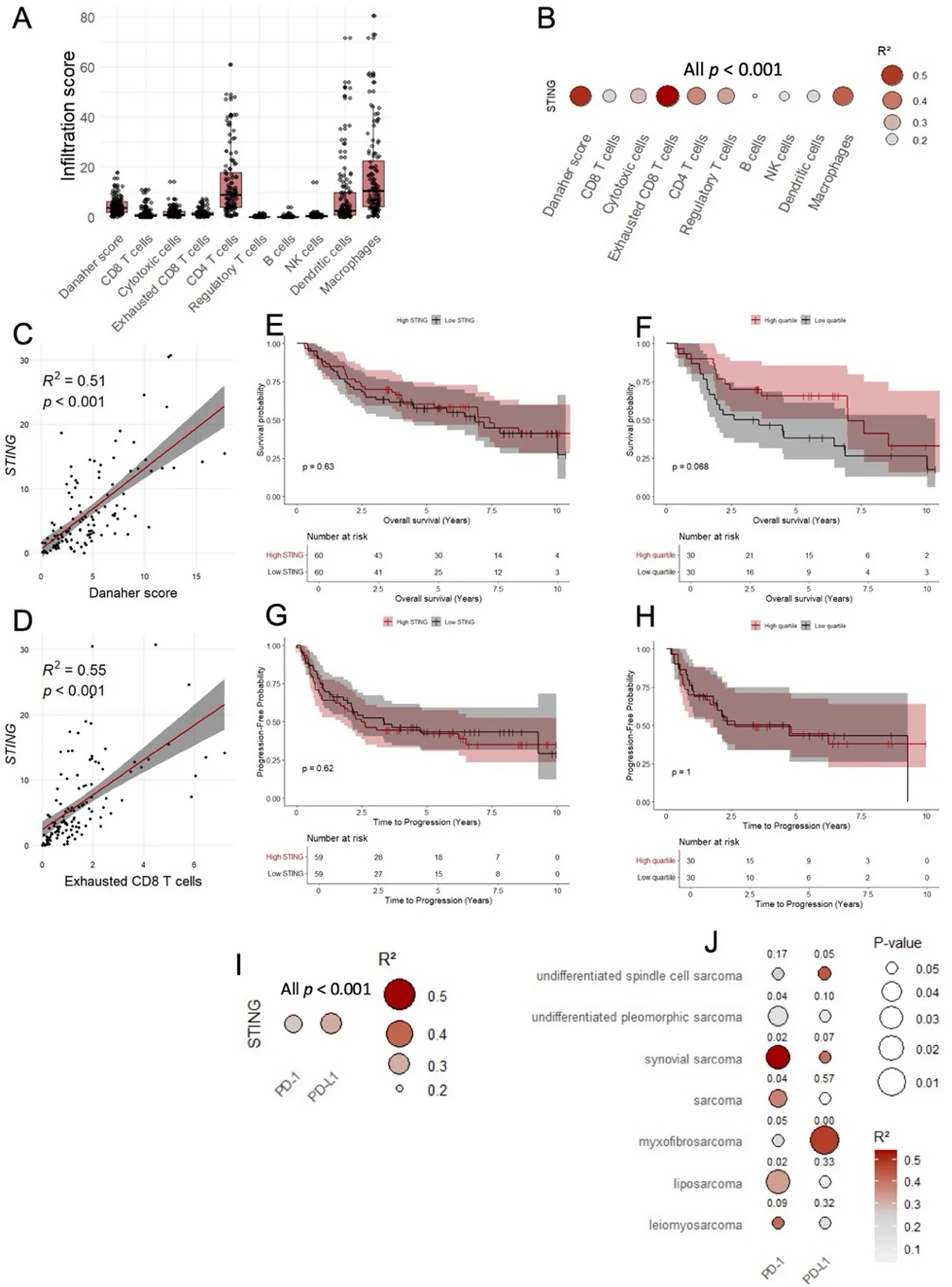
STING correlates with immune cell infiltration in a cohort of soft tissue sarcoma. **A** General immune infiltration score (Danaher score) and infiltration scores of indicated immune cell subsets in a cohort of 120 soft tissue sarcoma patients (the Aarhus STS cohort). Data were obtained from RNA sequencing. Median, 1st and 3rd quartiles are shown. **B** STING expression correlated with Danaher score and individual immune cell subtypes in the Aarhus cohort. The color and size indicate the *R*^2^-value of a spearman correlation. **C-D** Correlation plot of Danaher score and infiltration score for exhausted CD8 T with STING in the Aarhus cohort. Red line indicates a linear regression ± SEM. **E**–**H** Kaplan–Meier survival curves show the overall survival (OS) (**E–F**) and progression-free survival (PFS) (**G-H**) of soft tissue sarcoma patients from the Aarhus STS cohort divided by the median (**E** and **G**) or high and low quartiles (**F** and **H**) STING expression into a high STING (red) and low STING (black) group. The number of patients at risk in each group is displayed below the plot. A log-rank test was performed, and the resulting P-values are indicated. **I-J** STING expression correlated with PD-1 and PD-L1 expression in the Aarhus STS cohort for all patients (**I**) or in individual histological subtypes occurring > 4 times in the cohort (**J**). In **I** the color and size indicate the *R*^2^-value of a spearman correlation. In **J** the P-value is indicated above each data point, and the size indicates the P-value while the color indicates the *R*^2^-value of a spearman correlation

To examine STING expression and its relationship with immune infiltration and prognosis in STS, we performed bulk RNA sequencing on a large national STS cohort governed by Aarhus University Hospital. Patients with aggressive STS subtypes (*N* = 120) were selected, and immune infiltration was assessed using the Danaher deconvolution method [30]. STSs displayed a markedly heterogeneous immune landscape, with substantial variation in specific populations, particularly CD4 T cells, dendritic cells, and macrophages (Fig. 1A, Supplementary Table 1).

To evaluate whether poor ICI response in STS could be explained by low immune infiltration, pan-cancer immune infiltration across the Cancer Genome Atlas (TCGA) was compared [31] (Supplementary Fig. 1A–C). Using the same deconvolution approach, sarcoma (SARC) displayed consistently lower Danaher, CD8 T cell, and CD4 T-cell scores than nearly all tumor types for which PD-1/PD-L1-targeted ICI is approved (Supplementary Table 2). To consider intrinsic differences in tissue-specific immune environments, non-diseased tissues from GTEx were analyzed [32]. Tissues giving rise to ICI-responsive tumors (lung, bladder, breast, cervix, colon, esophagus, stomach, and uterus) generally ranked in the upper two-thirds of immune infiltration, whereas tissues commonly associated with STS showed substantial variability (Supplementary Fig. 1D). Notably, skeletal muscle exhibited among the lowest immune scores, while adipose tissue ranked among the highest.

Collectively, these findings show that immune infiltration in STS is highly heterogeneous and generally lower than observed in ICI-responsive cancers. This aligns with poor ICI responses in STS and highlights the complexity of tissue-derived immune microenvironments.

### STING expression correlates with immune cell infiltration in STS

Next, the association between STING expression and immune infiltration in STS was examined. In the Aarhus cohort, STING expression strongly correlated with overall immune infiltration, as measured by the Danaher score (*R*^2^ = 0.51, *P* < 0.001), and with an exhausted CD8 signature (*R*^2^ = 0.55, *P* < 0.001) (Fig. 1B–D). Significant correlations were also observed with macrophages (*R*^2^ = 0.44), CD4 T cells (*R*^2^ = 0.37), and regulatory T cells (*R*^2^ = 0.33) (Fig. 1B, Supplementary Fig. 2). These associations were independently validated in the TCGA SARC dataset (Supplementary Fig. 1E), indicating that STING expression robustly tracks immune-inflamed sarcomas across cohorts.

Consistent with prior reports, immune infiltration was associated with improved survival. TCGA SARC patients with high Danaher scores showed significantly longer survival (*P* = 0.013) (Supplementary Fig. 1F), while CD8 infiltration showed a favorable trend (*P* = 0.059) (Supplementary Fig. 1G). Despite its strong association with immune infiltration, STING expression alone in the Aarhus cohort did not associate with OS when stratified by the median (Fig. 1E). Quartile-based stratification revealed a trend toward improved OS in the upper STING quartile (*P* = 0.068) (Fig. 1F). No correlation with PFS was observed (Fig. 1G–H). Similarly, TCGA analyses showed no significant OS benefit except when comparing the highest versus lowest STING quartiles, in which a trend toward improved survival emerged (*P* = 0.052) (Supplementary Fig. 1H-I).

In the Aarhus cohort, STING expression did also correlate significantly with PD-L1 (*R*^2^ = 0.30, *P* < 0.001), whereas PD-1 fell slightly below the relevance threshold (*R*^2^ = 0.27) (Fig. 1I). Analysis by histological subtype (≥ 4 cases) revealed substantial variability. STING strongly correlated with PD-L1 in myxofibrosarcoma (MFS) (*R*^2^ = 0.48, *P* < 0.001), and strongly with PD-1 in synovial sarcoma (*R*^2^ = 0.54, *P* = 0.02), sarcoma NOS (*R*^2^ = 0.38, *P* = 0.04), and liposarcoma (*R*^2^ = 0.33, *P* = 0.02) (Fig. 1J).

Together, STING expression robustly defines immune-inflamed STS across independent cohorts (Aarhus and TCGA). While STING is not independently prognostic, very high expression is associated with improved survival and immune checkpoint expression in a subtype-specific manner, suggesting a functional role for STING in shaping the sarcoma immune microenvironment.

### STING expression is associated with cytokine production in STS

**Fig. 2:**
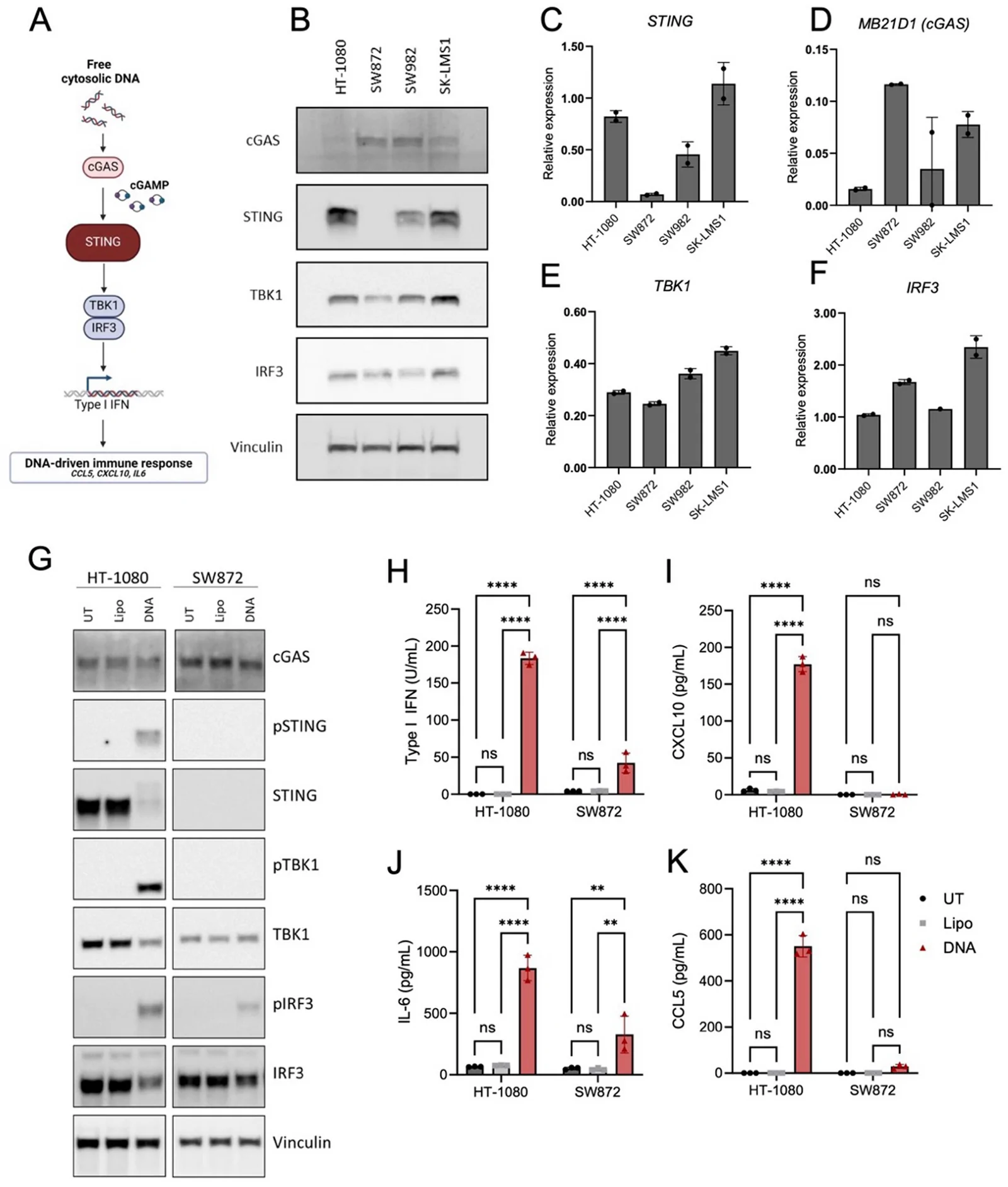
Cell line-dependent STING suppression hinders cytokine response to cytosolic DNA. **A** An illustration of essential elements of the STING pathway. **B** Expression of STING pathway proteins was evaluated using western blot in four soft tissue sarcoma cell lines. Vinculin was used as a loading control. **C**–**F** Gene expression of STING pathway genes obtained from RT-qPCR. Expression was normalized to EIF2B2. Mean ± standard deviation of two replicates is shown. **G** Expression and phosphorylation (p) of STING pathway proteins cGAS, pSTING(Ser366), total STING, pTBK1(Ser172), total TBK1, pIRF3(Ser386), and total IRF3 in SW872 and HT-1080 after transfection with herring testis DNA (DNA) (1 μg/mL) for 6 h using lipofectamine 2000 as transfection agent. A lipofectamine control (Lipo) and an untreated sample (UT) were included. Vinculin was used as loading control. **H**–**K** SW872 and HT-1080 cells were transfected for 24 h with DNA (1 μg/mL) using lipofectamine2000 as transfection agent, and supernatants were harvested for cytokine measurements of Type I IFN using a functional cell reporter assay and CXCL10, IL-6, and CCL5 using ELISA. A lipofectamine control (Lipo) and an untreated sample (UT) were included. Mean ± standard deviation of three replicates is shown. Two-way ANOVA analysis using Šídák’s multiple-comparisons test was performed; *p* < 0.05*, *p* < 0.01**, *p* < 0.001***, *p* < 0.0001****

To explore whether STING expression can be functionally linked to innate immune signaling in STS, we assessed STING pathway activity across four different human sarcoma cell lines. Protein and transcript evaluation confirmed expression of cGAS, TBK1, and IRF3 across all STS lines, whereas STING expression was absent in SW872 (Fig. 2A–F). Flow cytometric analysis of STING and PD-L1 confirmed reduced or absent expression of both markers in SW872 compared to HT-1080 (Supplementary Fig. 3A-B). Thus, STING-deficient SW872 and STING-proficient HT-1080 were selected as a paired model to evaluate pathway functionality.

Following dsDNA stimulation, STING-proficient HT-1080 exhibited phosphorylation of STING, TBK1, and IRF3 (Fig. 2G). This was accompanied by strong production of type I IFN, and inflammatory chemokines, CXCL10, IL-6, and CCL5 (Fig. 2H–K). In contrast, STING-deficient SW872 showed impaired phosphorylation and reduced cytokine responses, though minimal DNA-sensing activity remained.

To exclude a global defect in cytokine production, both cell lines were stimulated with poly(I:C), which activates RNA-sensing pathways independently of STING. Here, we detected robust cytokine responses (Supplementary Fig. 4A–D), confirming that SW872 had not lost the general ability to produce cytokines. These findings show that STING loss impairs the response to cytosolic DNA in STS cells.

### STING functionality is restored with CRISPR-mediated transcriptional activation

**Fig. 3:**
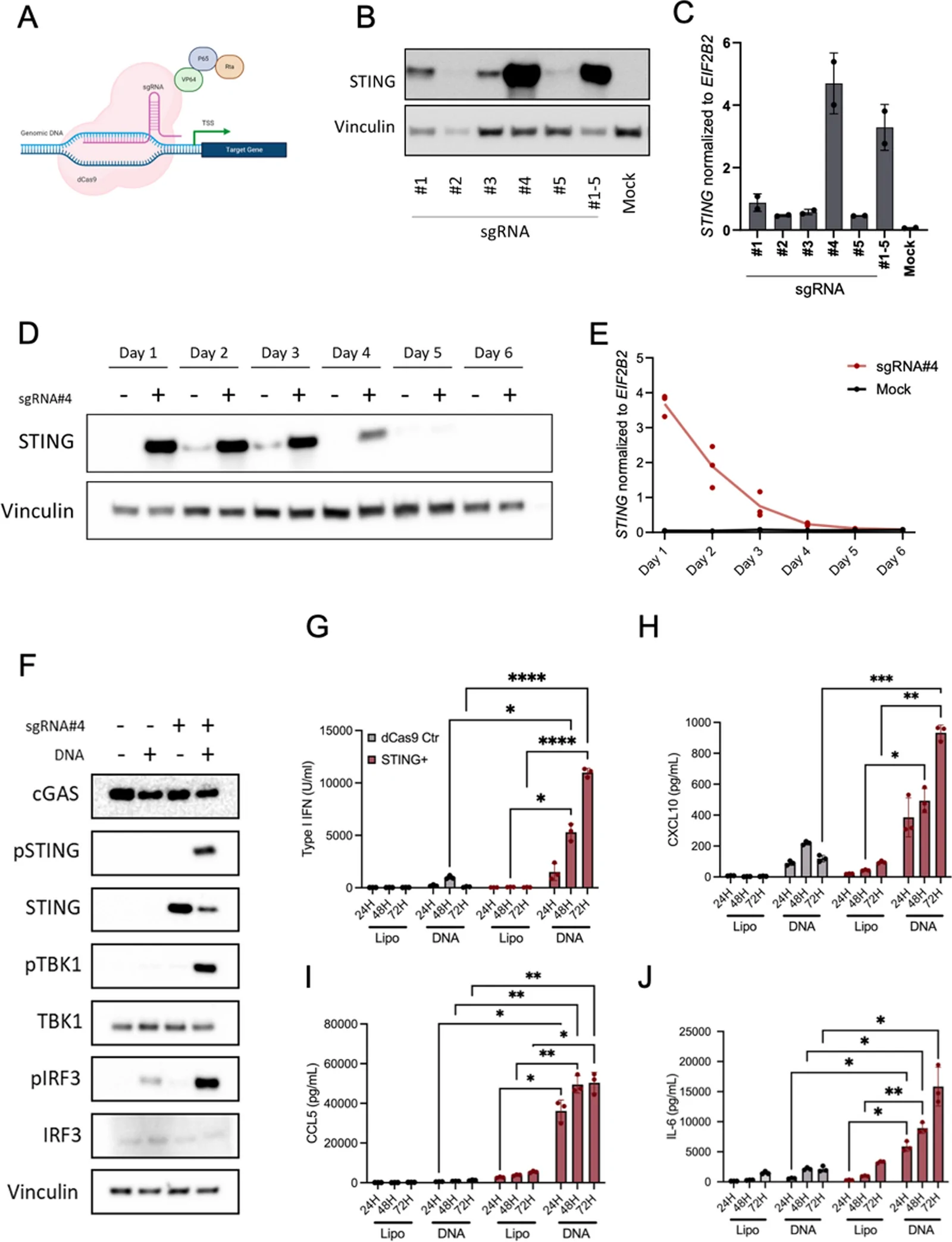
STING expression and functionality are restored with CRISPR-mediated transcriptional activation. **A** CRISPR-mediated transcriptional activation (CRISPRa) complex consisting of deactivated Cas9 nuclease (dCas9), guide RNA (sgRNA), and transcription factors VP64, p65, and Rta (VPR), binding to the promoter region of the target gene. **B-C** STING protein expression **B** and mRNA expression **C** were evaluated using western blot and RT-qPCR in SW872 24 h after performing CRISPRa of STING using different sgRNAs (#1, #2, #3, #4, #5, or #1-#5 in combination). A mock control without CRISPRa was included. Vinculin was used as loading control in (**B**) and EIF2B2 as reference gene in (**C**). Mean ± standard deviation of two replicates is shown in (**C**). **D-E** STING protein expression **D** or mRNA expression **E** were evaluated using western blot and RT-qPCR in SW872 after performing CRISPRa of STING using sgRNA#4 for the indicated number of days. A mock control without CRISPRa was included for each day. Vinculin was used as loading control in (**D**) and EIF2B2 as reference gene in (**E**) Mean ± standard deviation of three replicates is shown in (**E**). **F** CRISPRa for STING was performed in SW872 using sgRNA#4 ( +) one day prior to transfection with herring testis DNA (DNA) (2 μg/mL) for 6 h using lipofectamine2000 as transfection agent ( +). Expression and phosphorylation(p) of STING pathway proteins cGAS, pSTING(Ser366), total STING, pTBK1(Ser172), total TBK1, pIRF3(Ser386), and total IRF3 were evaluated using western blot. A mock control without CRISPRa (-) and an untreated control (-) were included. Vinculin was used as loading control. **G-J** SW872 cells exposed to CRISPR activation were collected at 24, 48, or 72 h and transfected with herring testis DNA (DNA) (2 μg/mL) for 24 h using lipofectamine2000 as transfection agent. A mock control with no CRISPRa modulation was included. Supernatants were then harvested for cytokine measurements of Type I IFN using a functional cell reporter assay and CXCL10, CCL5, and IL-6 using ELISA. Mean ± standard deviation of three replicates is shown. Two-way ANOVA analysis using Tukey multiple-comparisons test was performed; *p* < 0.05*, *p* < 0.01**, *p* < 0.001***, *p* < 0.0001****

To test whether STING-controlled epigenetic reexpression could restore DNA-induced cytokine signaling, CRISPR activation (CRISPRa) targeting the STING promoter using dCas9-VPR was applied (Fig. 3A). Screening of five sgRNAs individually and in combination identified strong STING induction at both protein and mRNA levels in STING-deficient SW872 cells (Fig. 3B–C). A time-course analysis further demonstrated STING upregulation lasting up to four days—determined both on protein and RNA expression levels (Fig. 3D–E).

Next, we evaluated the functional effects of CRISPRa-induced STING expression and whether this restored activation of the dsDNA-sensing pathway. Twenty-four hours after STING restoration in SW872 cells, DNA stimulation induced phosphorylation of STING, TBK1, and IRF3, indicating reactivation of downstream signaling (Fig. 3F). Next, to assess the durability of pathway reactivation, DNA stimulation was repeated at multiple days following CRISPRa-induced STING expression. Here, we could confirm both elevated STING protein expression (Supplementary Fig. 5A-B) and prolonged cGAS-STING pathway activity, resulting in significantly elevated induction of type I IFN and proinflammatory cytokines at all time points (Fig. 3G–J).

Together, these results demonstrate that epigenetic or transcriptional silencing of STING is reversible by CRISPRa-mediated activation of endogenous STING, which restores not only its expression but also functional STING signaling in STS cells.

### STING activation through LNPs in patient-derived sarcoma cells triggers HLA class I-dependent T-cell recognition

**Fig. 4:**
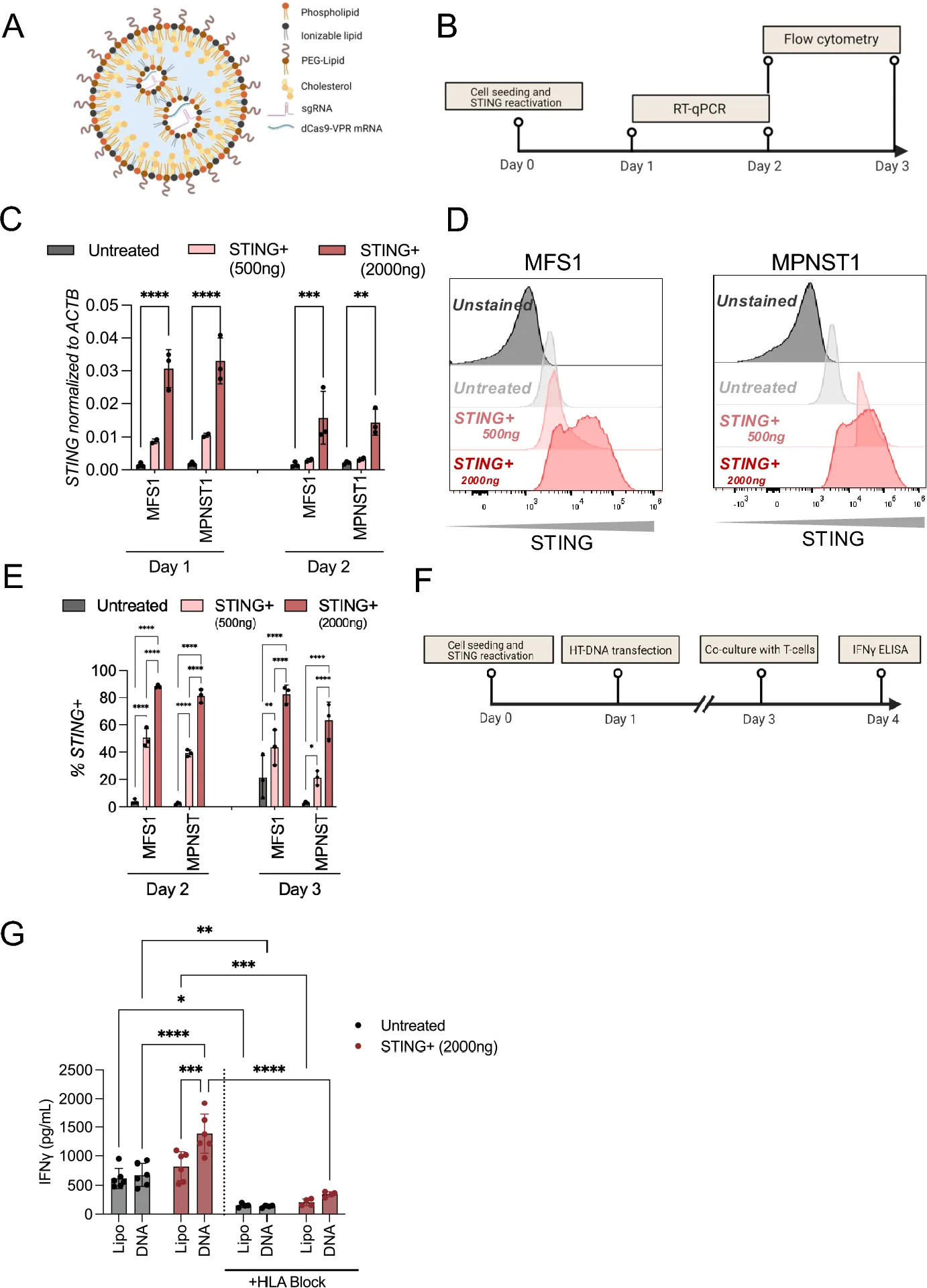
STING activation through LNPs in ex vivo patient-derived sarcoma cells triggers HLA class I-dependent T-cell recognition. **A** An illustration of a LNP used for transcriptional upregulation of STING. **B** The experiment timeline for data in (**C–E**). CRISPRa of STING (STING+) with LNP delivery was performed in MFS1 and MPNST1 using 500 ng or 2000 ng of sgRNA and dCas9-VPR mRNA per 200,000 cells seeded and evaluated by **C** STING mRNA expression day 1 and 2 after CRISPRa (**C**); STING protein expression (**D**) and percentage of STING positive cells (% STING +) (E). For (D-E) data were measured using flow cytometry day 2 and 3 after CRISPRa. An untreated control (**C**) and an unstained control **D-E** stained for STING were included. ACTB was used as a reference gene in (**C**). A Two-way ANOVA analysis using Sídak’s multiple-comparisons test was performed in (**C** and **E**). *p* < 0.05*, *p* < 0.01**, *p* < 0.001***, *p* < 0.0001****. **F** The experiment timeline for data in (**G**). **G** CRISPRa of STING (STING+) with LNP delivery using 2000 ng of sgRNA and dCas9-VPR mRNA per 200,000 cells was performed one day prior to transfection with herring testis DNA (DNA) (2 μg/mL) for 6 h whereafter the cell culture media was changed. Two days after transfection, tumor cells were co-cultured with T cells for 24 h with or without an HLA blocking antibody. An untreated control that was not subjected to CRISPRa and a lipofectamine control (Lipo) was included. Replicates from three independent experiments are pooled and plotted with mean ± standard deviation indicated. Two-way ANOVA analysis using Turkey’s multiple-comparisons test is performed. *p* < 0.05*, *p* < 0.01**, *p* < 0.001***, *p* < 0.0001****

To extend these findings beyond established cell lines, LNP delivery of CRISPRa components was evaluated in two patient-derived ex vivo cultures of myxofibrosarcoma and malignant peripheral nerve sheath tumor (MFS1 and MPNST1, respectively) (Fig. 4A-B). Treatment with LNP-CRISPRa STING significantly upregulated STING RNA and protein levels (Fig. 4C–E, Supplementary Fig. 6), confirming effective delivery and transcriptional activation.

Next, the functional consequences for T-cell recognition were assessed using autologous tumor-infiltrating lymphocytes (TILs). Enhanced tumor recognition, measured by IFNγ release, was observed in cell cultures exposed to LNP-CRISPRa STING followed by dsDNA stimulation (Fig. 4F–G). HLA class I blockade abrogated this recognition, confirming that T-cell recognition was HLA class I dependent. To further characterize these observations, we assessed HLA class I and PD-L1 expression. STING restoration increased HLA class I expression upon dsDNA stimulation compared with untreated or unstimulated controls (Supplementary Fig. 7A). In contrast, PD-L1 expression was reduced following STING restoration under baseline conditions, while dsDNA transfection induced a modest increase in PD-L1 levels irrespective of STING activation (Supplementary Fig. 7B).

To determine whether STING restoration induced downstream transcriptional changes in the absence of exogenous stimulation, we assessed multiple gene expression levels three days post LNP-CRISRPa delivery. Interestingly, both STING and CXCL10 were significantly upregulated, whereas IFNB1, PD-L1, or VIPERIN was not (Supplementary Fig. 8A–E). This suggests that endogenous cytosolic DNA in STS cells may activate the pathway once STING is restored.

Overall, LNP-CRISPRa mediated STING induction sensitized patient-derived tumor cells to CD8 T-cell recognition via HLA class I.

### STING activation in sarcoma patient-derived spheroids triggers T-cell-mediated killing

**Fig. 5:**
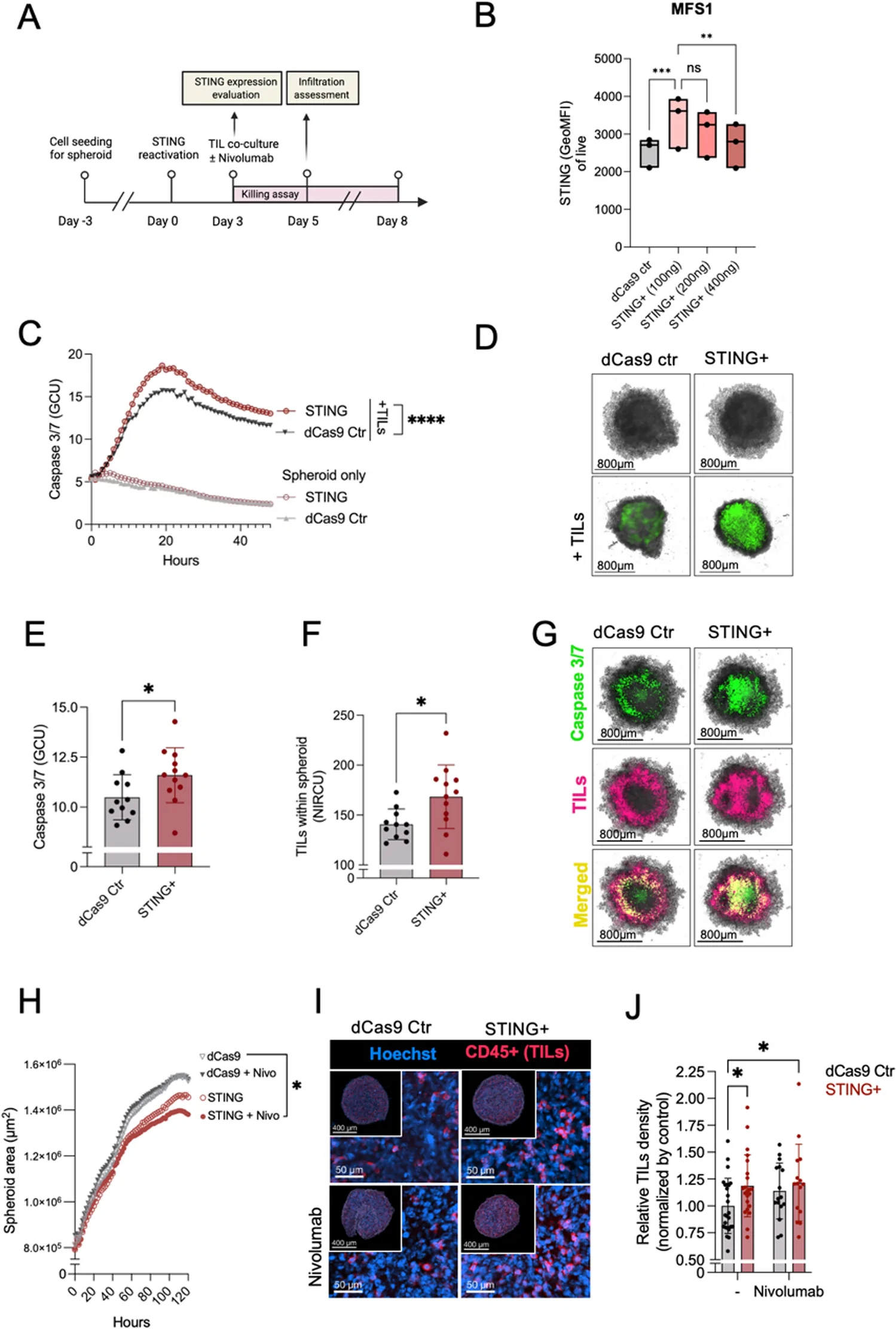
STING activation in ex vivo sarcoma patient-derived spheroids triggers T-cell-mediated killing. **A** The experiment timeline for data in (**B–E)** Created using biorender.com. **B** CRISPRa of STING (STING+) using LNP delivery was performed on established tumor spheroids of MFS1 cells with the indicated concentrations of guide RNA (sgRNA) and dCas9-VPR mRNA (100–400 ng). STING expression was evaluated on day 3 using flow cytometry and plotted as geometric mean fluorescence intensity (GeoMFI). A control with 400 ng of dCas9-VPR mRNA per 200,000 cells seeded was included (dCas9 ctr). Mean ± standard deviation of three individual experiments is shown. Two-way ANOVA analysis using Dunnett’s multiple-comparisons test is performed. **C**-**D** CRISPRa of STING (STING +) using LNP delivery was performed on established tumor spheroids of MFS1 cells. On day three after CRISPRa, a 48-h co-culture of tumor spheroids and autologous tumor-infiltrating lymphocytes (TILs) was initiated. Killing of tumor spheroids was measured by caspase 3/7 activation as green calibrated units (GCU). **C** Quantification of caspase activation in spheroids. Mean of 12 replicates is plotted. RM Two-way ANOVA analysis is performed. **D** Representative images at 24 h of TILs-spheroid co-culture displaying caspase activation. **E** Caspase 3/7 quantification (GCU) within spheroids in co-culture with autologous TILs at 24 h. **F** Quantification of labeled TILs as near-infrared calibrated units (NIRCU) within spheroid at 24 h. **G** Representative images of Caspase 3/7 Green, TILs (Pink), and merged (yellow) depicting spheroid infiltration, killing and co-localization at 24 h. (H) Assessment of tumor–immune control in 3D spheroids generated from MFS1 cells co-cultured with patient-derived TILs following STING reactivation and/or PD-1 blockade (Nivolumab, 25 µg/mL). Spheroid growth was quantified over time by measuring spheroid area (µm^2^). **I** Representative images of 3D-reconstructed confocal images staining for CD45 (TILs). Whole spheroids are shown, with higher-magnification views of the spheroid core to illustrate TIL distribution and density. **J** Quantification of TIL infiltration within spheroids based on 3D reconstruction analysis. Each dot represents an individual spheroid. TIL infiltration is shown relative to the dCas9 control condition. One-way ANOVA was used in (**B)**. Repeated-measures two-way ANOVA, including treatment and time interaction, was used to assess differences between GCU and growth curves in **C** and **H**. Unpaired Student *t*-test is used for (**H)** and two-way ANOVA for (**J)**. *p* < 0.05*, *p* < 0.01**, *p* < 0.001***, *p* < 0.0001****

To extend our findings to a physiologically relevant setting, 3D ex vivo spheroid cultures from MFS1 were used (Fig. 5A). Here, we found that lower sgRNA and dCas9-VPR doses were sufficient to induce STING expression in a MFS1 spheroids 3D architecture (Fig. 5B).

To assess functional consequences, patient-derived spheroids were next co-cultured with autologous TILs (Fig. 5A). STING reactivation significantly increased T-cell-mediated killing (Fig. 5C–E), accompanied by enhanced TIL infiltration and spatial co-localization with caspase 3/7 within the spheroid (Fig. 5F-G), although the frequency of TILs was not significantly different by flow cytometry following spheroid dissociation (Supplementary Fig. 9A-C). Consistent with this, HLA Class I expression was also upregulated in the presence of TILs compared to monoculture (-TILs) and significantly increased upon STING restoration (Supplementary Fig. 10A). This effect was independent of the LNPs alone (Supplementary Fig. 10B). In contrast, PD-L1, although downregulated by STING restoration in monoculture, was significantly increased in the presence of TILs, with a minimal increase in STING-restored conditions (Supplementary Fig. 10C).

Based on these observations, we wanted to assess the combination of STING restoration with PD-1 blockade (Nivolumab). While STING restoration alone showed a trend toward TIL-mediated improved tumor control, the combination with Nivolumab resulted in a significant reduction in spheroid growth over time compared to untreated controls (Fig. 5H). Consistently, 3D confocal reconstruction revealed increased TIL abundance within spheroids upon STING restoration (Fig. 5I–J).

These findings demonstrate that LNP-CRISPRa-mediated STING restoration promoted T-cell-mediated cytotoxicity in patient-derived sarcoma models. This is further supported by increased TIL infiltration and spatial association with tumor cell apoptosis, and by improved tumor control with PD-1 blockade.

## Discussion

In this study, we identify an association between the STING-controlled innate immune pathway and antitumor immunity in STS. In tumor tissues from our newly established clinical cohort of 120 patients with STS, immune infiltration was positively associated with STING expression, a finding further confirmed in the TCGA sarcoma cohort of 265 patients. We demonstrate that CRISPRa-mediated epigenetic restoration of STING expression in patient-derived sarcoma 3D models enhances the activation of patient-derived T cells. Importantly, this strategy can be implemented using LNPs-mediated delivery, highlighting its therapeutic potential.

The STING pathway is central to innate immune signaling and has been implicated in cancer immune evasion. Although STING loss facilitates immune escape, chronic STING activation may promote tumor progression and metastasis through type I IFN tachyphylaxis or NF-κB signaling, particularly in the context of chromosomal instability [33–35]. Thus, precise modulation of STING activity is essential. By transiently restoring STING expression using LNP-delivery CRISPRa, we avoid chronic activation and downstream tumor-promoting effects. This strategy may prime the TME for subsequent treatments, including radiotherapy, chemotherapy, and PD-1/PD-L1 ICI therapy. Other nanoparticle delivery approaches have recently been suggested including molecular structures delivering or releasing metals, DNA, or toxins specifically to the tumor and thereby trigger activation of cGAS-STING [36–38]. However, such approaches still depend on the presence of STING, suggesting that a future combination of nanoparticles both elevating STING expression and triggering stronger STING activation may be applicable.

To date, most therapeutic efforts have focused on developing STING agonists. However, our findings suggest that STING agonist therapy alone may be ineffective in STS, particularly in tumors with insufficient STING expression. Preclinical studies have shown that STING agonists can synergize with PD-1/PD-L1 blockade in cervical cancer [39] and that a STING agonist–anti-PD-L1 conjugate effectively inhibited tumor growth across several cancers [40]. The small-molecule agonist MSA-2, combined with PD-L1 knockout, further increased type I IFN signaling and cytotoxic lymphocyte activity [41]. However, little is known about how STING influences immunotherapy responses in STS, owing to the rarity and heterogeneity of these tumors.

STS subtypes can be broadly classified into those with simple karyotypes (e.g., synovial sarcoma) and those with complex karyotypes (e.g., UPS, leiomyosarcoma, dedifferentiated liposarcoma) [4, 9]. In SARC028, UPS and DDLPS responded favorably to PD-1 inhibitors, whereas synovial sarcoma did not (NCT00140855), and leiomyosarcoma showed poor outcomes in both the SARC028 and Alliance A091401 trials. In our cohort, we observed a strong positive correlation between PD-L1 and STING expression, particularly in MFS, a complex karyotype STS. This association may reflect the high degree of chromosomal instability in MFS, which could promote STING activation and PD-L1 transcription. However, in our ex vivo model, STING reactivation did not significantly upregulate PD-L1 in monoculture, although PD-L1 expression increased slightly in the presence of TILs following STING reactivation. Notably, muscle tissue exhibited low immune infiltration, suggesting tissue-of-origin effects may influence responses to ICI responses.

Although STING expression correlated with overall and specific immune infiltration, it did not correlate with OS or PFS. This may reflect the low mutational burden of STS where the absence of cytosolic DNA (generated from genomic instability) is insufficient to drive antitumor immunity though STING is expressed. In our 2D patient-derived sarcoma cell systems, however, STING activation did enhance TIL responses. Furthermore, in 3D spheroids, STING reexpression alone increased tumor killing, possibly owing to endogenous DNA derived from the necrotic core [42].

From a translational standpoint, our findings suggest that transient STING restoration using LNP‑delivered CRISPRa may represent a feasible therapeutic strategy for STS, particularly for tumors with epigenetic STING suppression. This approach leverages clinically advancing LNP technologies while avoiding the risks associated with permanent gene modification or chronic pathway activation. Still, several limitations remain. STS heterogeneity may influence which subtypes benefit most, and efficient in vivo delivery into dense sarcoma tissues has not yet been established. Furthermore, the safety, durability, and immune consequences of repeated CRISPRa dosing require careful evaluation, as excessive STING signaling can promote inflammation or tumor‑supportive pathways. Thus, future studies using in vivo cancer models are warranted to validate both the therapeutic efficacy and delivery specificity of LNP-CRISPRa.

Across cancer types, a consistent axis emerges in which genomic instability triggers STING activation, leading to immune infiltration and altered PD-1/PD-L1 expression, ultimately enhancing ICI responses [13, 20, 21, 43–46]. Because current therapies already induce genomic instability and PD-1/PD-L1 blockade is clinically available, targeted transcriptional activation of STING via LNP-CRISPRa may complete this axis in STS with suppressed STING expression. By restoring STING expression in these tumors, this strategy could help convert immunologically “cold” tumors into “hot” tumors and thereby improve patient outcomes.

## Supplementary Information

Below is the link to the electronic supplementary material.

Supplementary file1 (PDF 1238 kb)

Supplementary file2 (DOCX 22 kb)

## Data Availability

The datasets in this study are not publicly available. This is in accordance with the rules governing the processing of personal data, as described in the EU General Data Protection Regulation (GDPR) and the Danish Data Protection Act. However, should a researcher be interested in our data, they are welcome to contact Ninna Aggerholm-Pedersen, Department of Oncology, Aarhus University Hospital, DK.
