# Supplementary material for: Restoring STING expression in soft tissue sarcoma increases activation and function of tumor-infiltrating lymphocytes
Source: Cancer Immunol Immunother. 2026 Jun 21;75(10):227. doi: 10.1007/s00262-026-04455-3 (PMC13578154; doi:10.1007/s00262-026-04455-3)
Supplement: Supplementary file 1 [file 262_2026_4455_MOESM1_ESM.pdf]

Figure S1. Godsk et al.

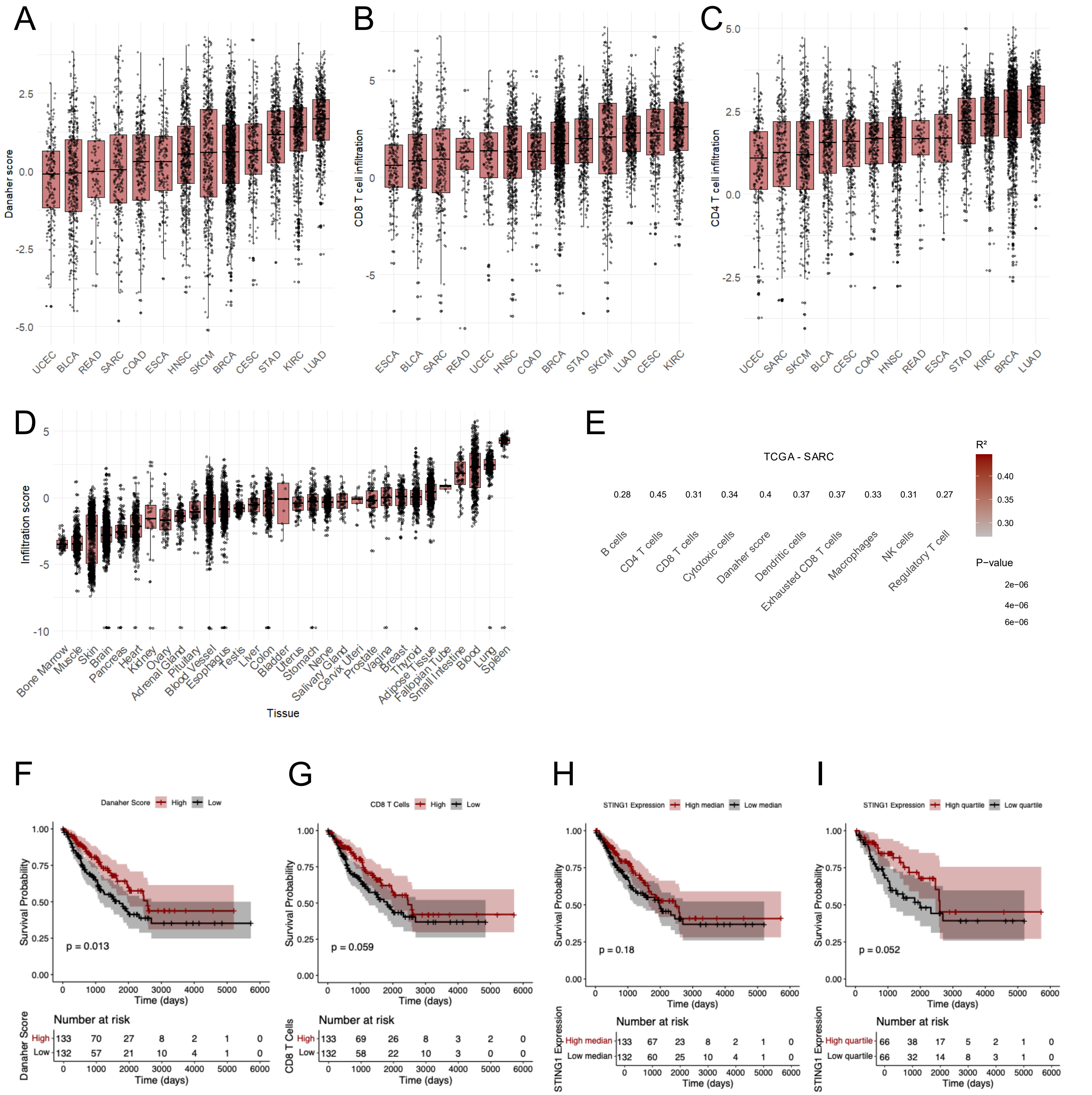

Supplementary Figure 1. Pan-cancer and tissue immune cell infiltration

(**A-C**) General immune infiltration score (Danaher score) (A) and infiltration scores of CD8 T cells (B) and CD4 T cells (C) in selected solid tumors in which immune checkpoint inhibitors targeting PD-1/PD-L1 are approved. Data is obtained from TCGA pan-cancer gene expression data. (**D**) General immune infiltration in healthy tissue of the indicated origin. Data is obtained from the GTEx portal. Data is scaled and median, 1st and 3rd quartiles are shown. (**E**) *STING* expression correlated to Danaher score and individual immune cell subtypes in the TCGA-SARC cohort. The color indicates the R^2-value of a Spearman correlation. The size of the sphere indicates the p-value. (**F-I**) Kaplan-Meier survival curves show the overall survival (OS) of soft tissue sarcoma patients from the SARC TCGA cohort divided by the median expression of Danaher Score (F), CD8 T cell score (G), and *STING* (H). In (I), patients are divided by upper and lower quartiles of *STING* expression. Red defines high groups, while black represents low expression. The number of patients at risk in each group is displayed below each plot.

Figure S2. Godsk et al.

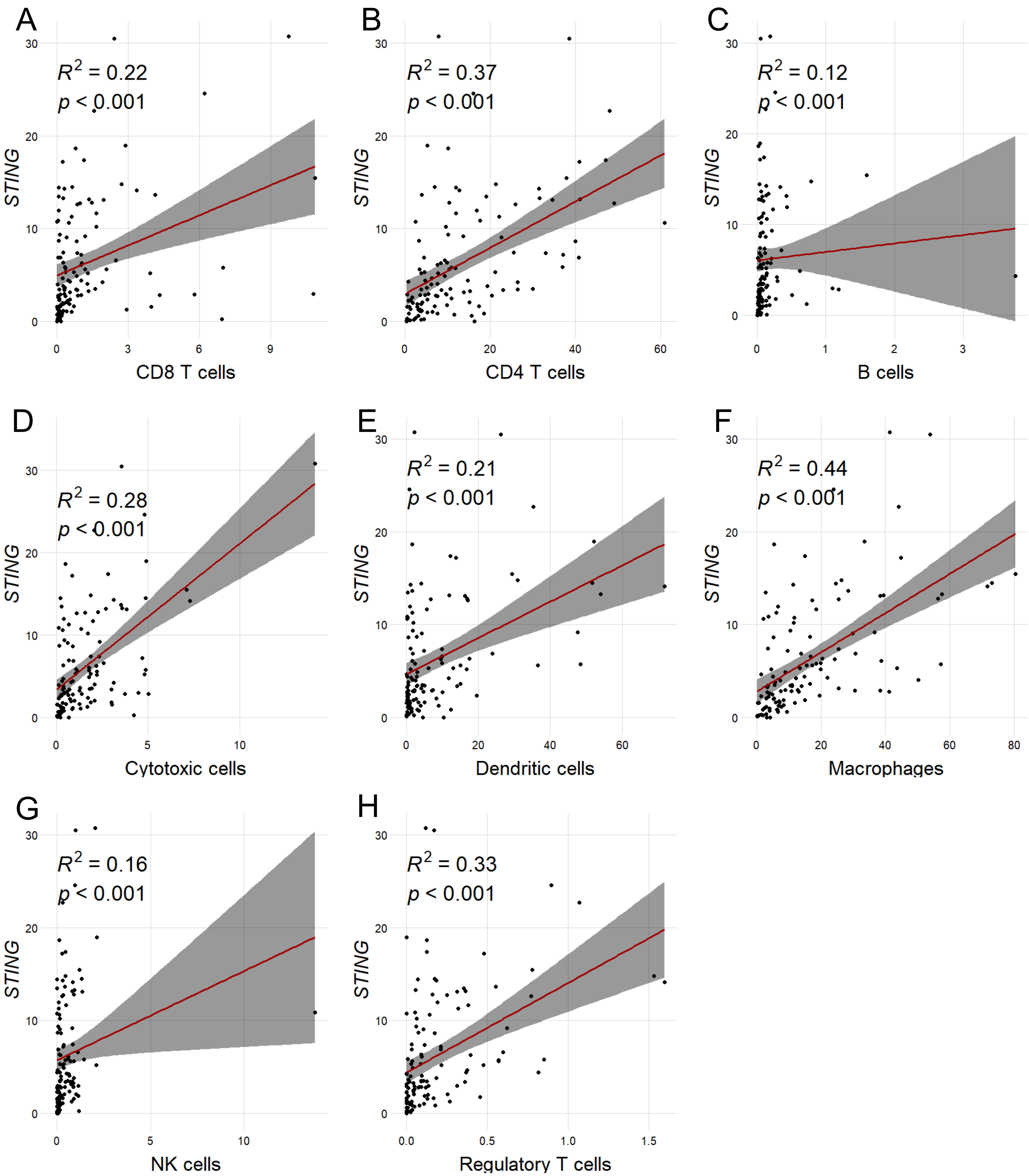

Supplementary Figure 2. STING expression correlates with immune cell infiltration

(**A-H**) *STING* expression correlated to individual immune cell subtypes infiltration. Data is obtained from RNA sequencing of a cohort of 120 soft tissue sarcoma patients. Red line indicates a linear regression +/- SEM. R^2- and p-values are obtained from a spearman correlation.

Figure S3. Godsk et al.

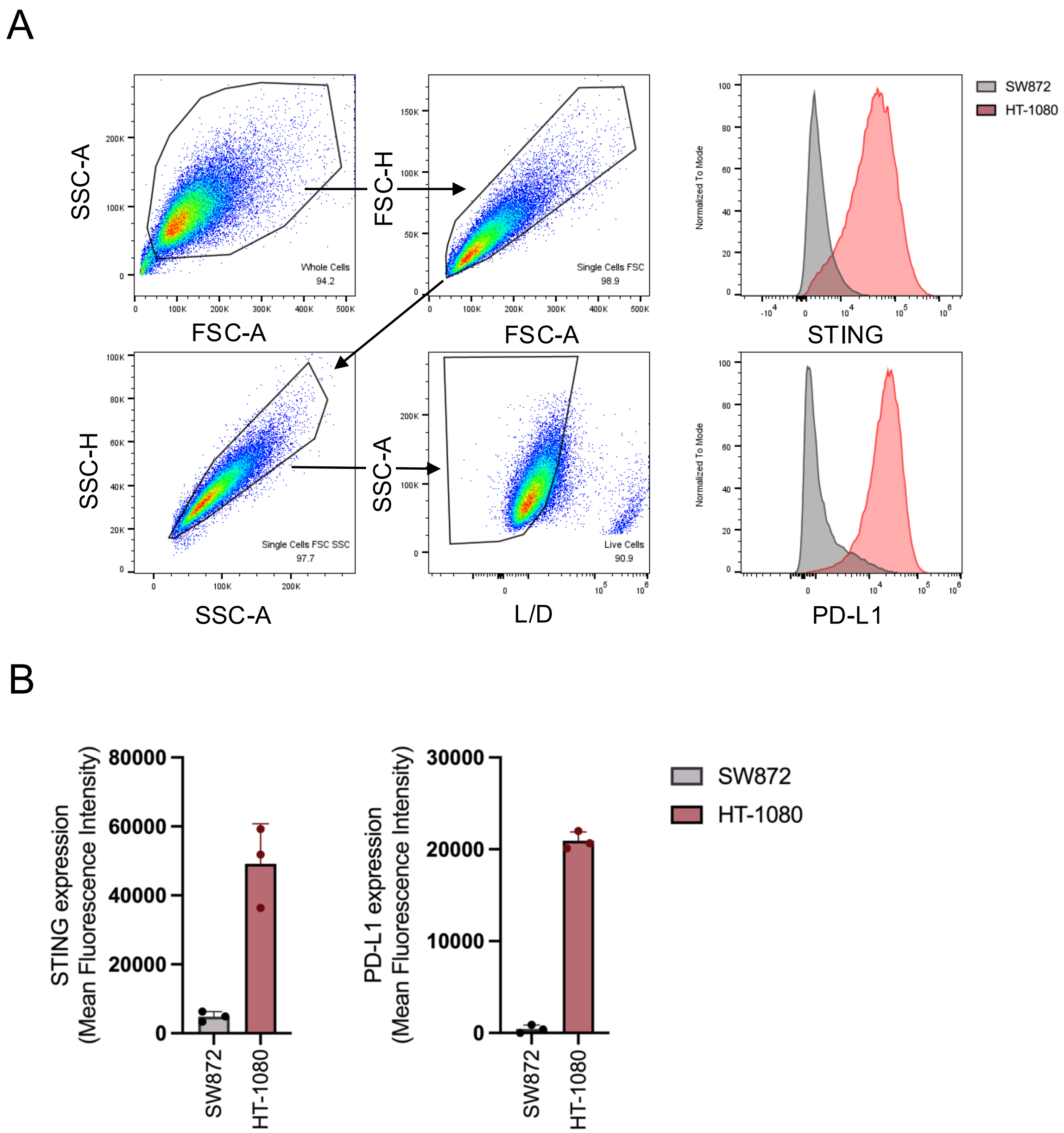

Supplementary Figure 3. STING and PD-L1 expression.

(**A**) SW872 and HT-1080 cells were cultured for at least 1 week followed by flow cytometry measurement of intracellular STING expression and surface expression of PD-L1. (**B**) Quantification (MFI) of STING and PD-L1 expression was done in three biological replicates of each cell line.

Figure S4. Godsk et al.

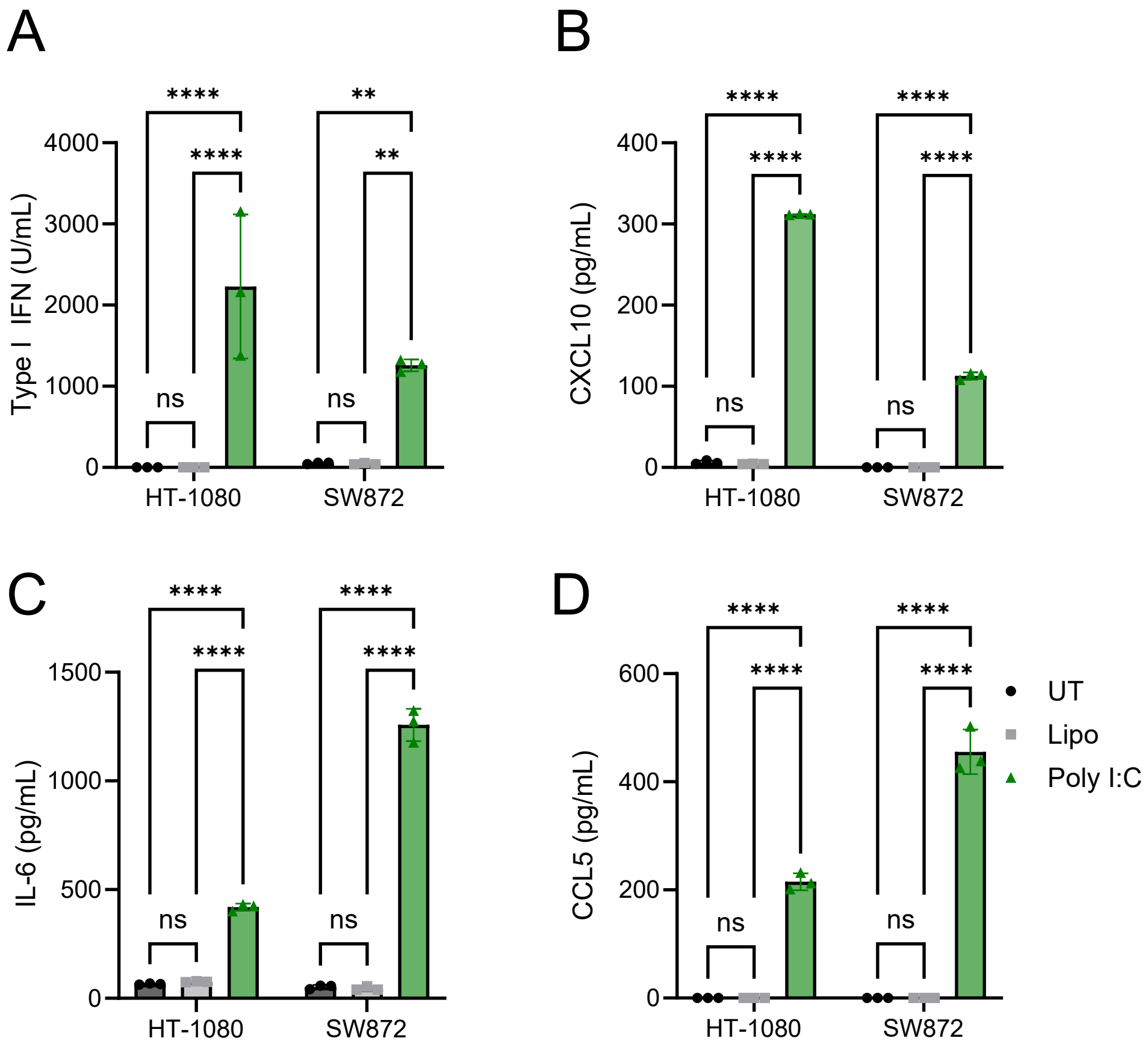

Supplementary Figure 4. Cytokines are expressed in response to Poly I:C

(**A-D**) SW872 and HT-1080 cells were transfected for 24 hours with DNA (1µg/mL) using lipofectamine2000 as transfection agent, and supernatants were harvested for cytokine measurements of Type I IFN using a functional cell reporter assay and CXCL10, IL-6, and CCL5 using ELISA. A lipofectamine control (Lipo) and an untreated sample (UT) was included. Mean +/- standard deviation of three replicates is shown. Two-way ANOVA analysis using Šídák's multiple comparisons test was performed; $p < 0.05$ *, $p < 0.01$ **, $p < 0.001$ ***, $p < 0.0001$ ****.

Figure S5. Godsk et al.

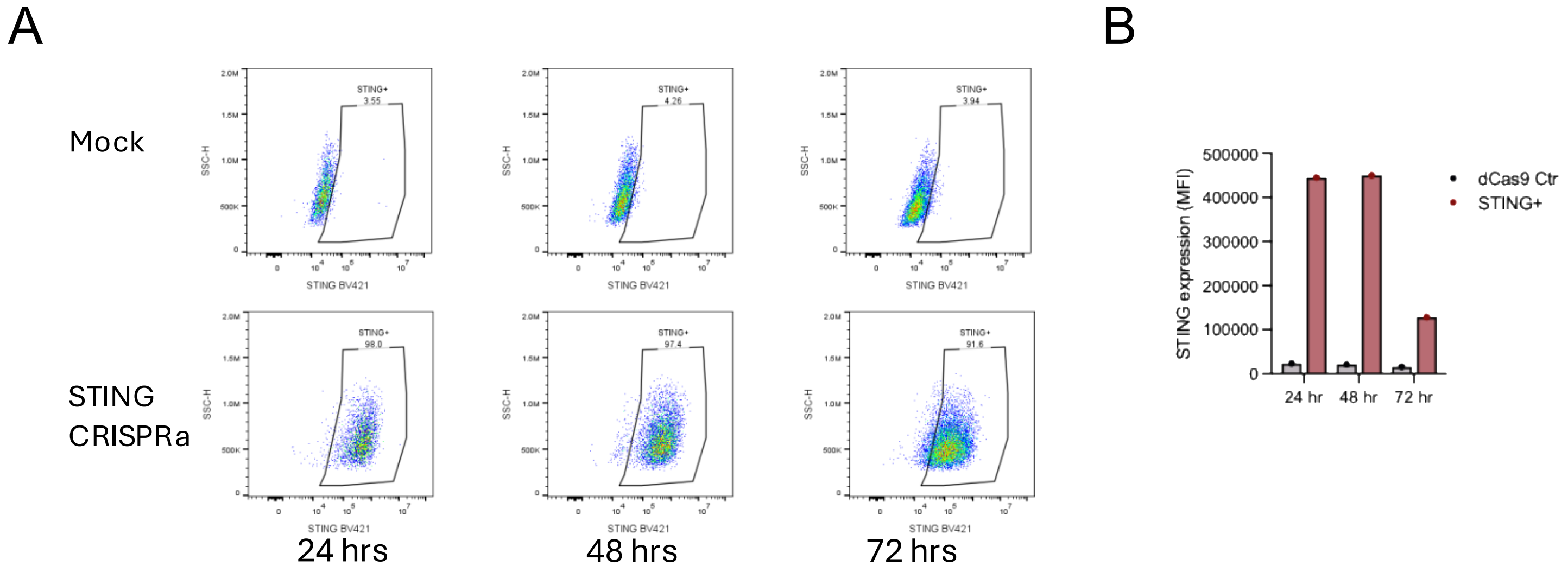

Supplementary Figure 5. STING expression and functionality is restored with CRISPR-mediated transcriptional activation over three days. (A-B) CRISPR-mediated transcriptional activation (CRISPRa) with sgRNA#4 was performed in SW872 cells. STING protein expression was evaluated by intracellular flow cytometry **(A)** and quantified 24, 48 and 72 hrs after CRISPR activation **(B)**.

Figure S6. Godsk et al.

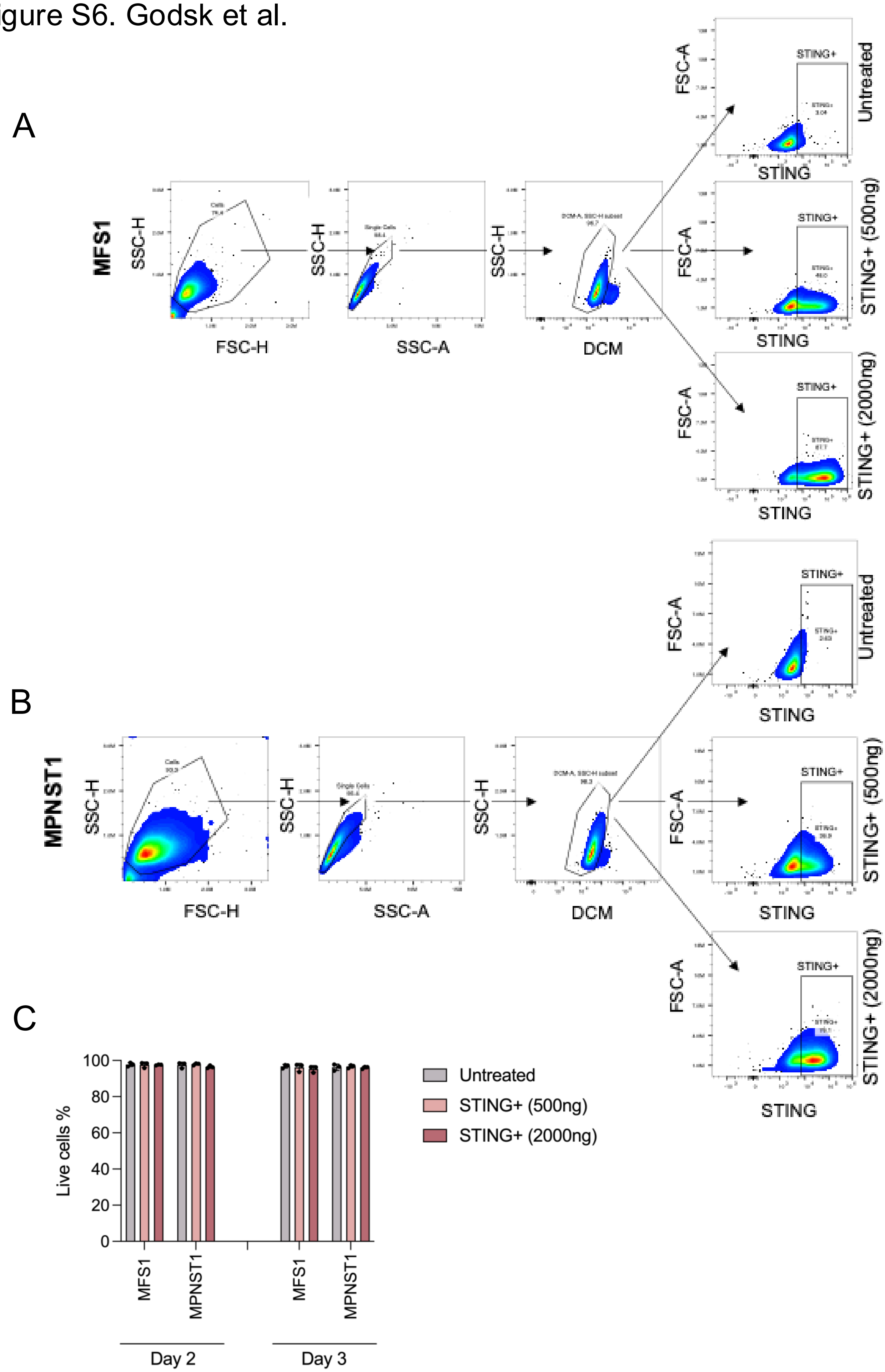

Supplementary Figure 6. Gating strategy MFS1 and MPNST1

(A-B) Tumor cells from MFS1 and MPNST1 patients were selected by Forward Scatter Height (FSC-H) and Side Scatter height (SSC-H). Using SSC height versus area, the singlets were gated, following the selection of dead cell marker (DCM)-negative cells as our live population. From the gated live cells, STING-positive cells were gated using unstained compensated controls and Untreated control. **(C)** Frequency of live patient-derived cells from MFS1 and MPNST1 2D cultures analyzed by flow cytometry 2 and 3 days post LNP delivery.

Figure S7. Godsk et al.

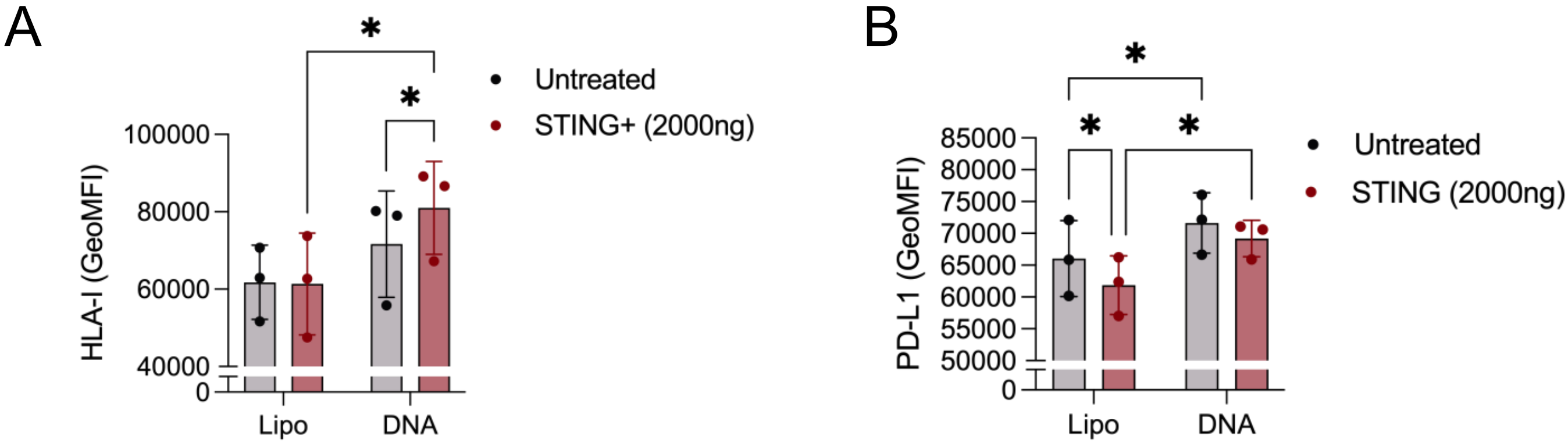

Supplementary Figure 7. HLA-I and PD-L1 expression in 2D MFS1 patient cultures upon STING restoration and dsDNA stimulation. (**A**) HLA-I expression in MFS1 patient-derived cells cultured in 2D and treated with sucrose (untreated) or STING+ LNPs (2000 ng). Stimulation was done with lipofectamine 2000 (Lipo) and HT-DNA in lipofectamine (DNA). (**B**) PD-L1 expression in MFS1 cells cultured in 2D following transfection with or without DNA . Two-way ANOVA was employed in A and B; $p < 0.05$ *, $p < 0.01$ **, $p < 0.001$ ***, $p < 0.0001$ ****.

Figure S8. Godsk et al.

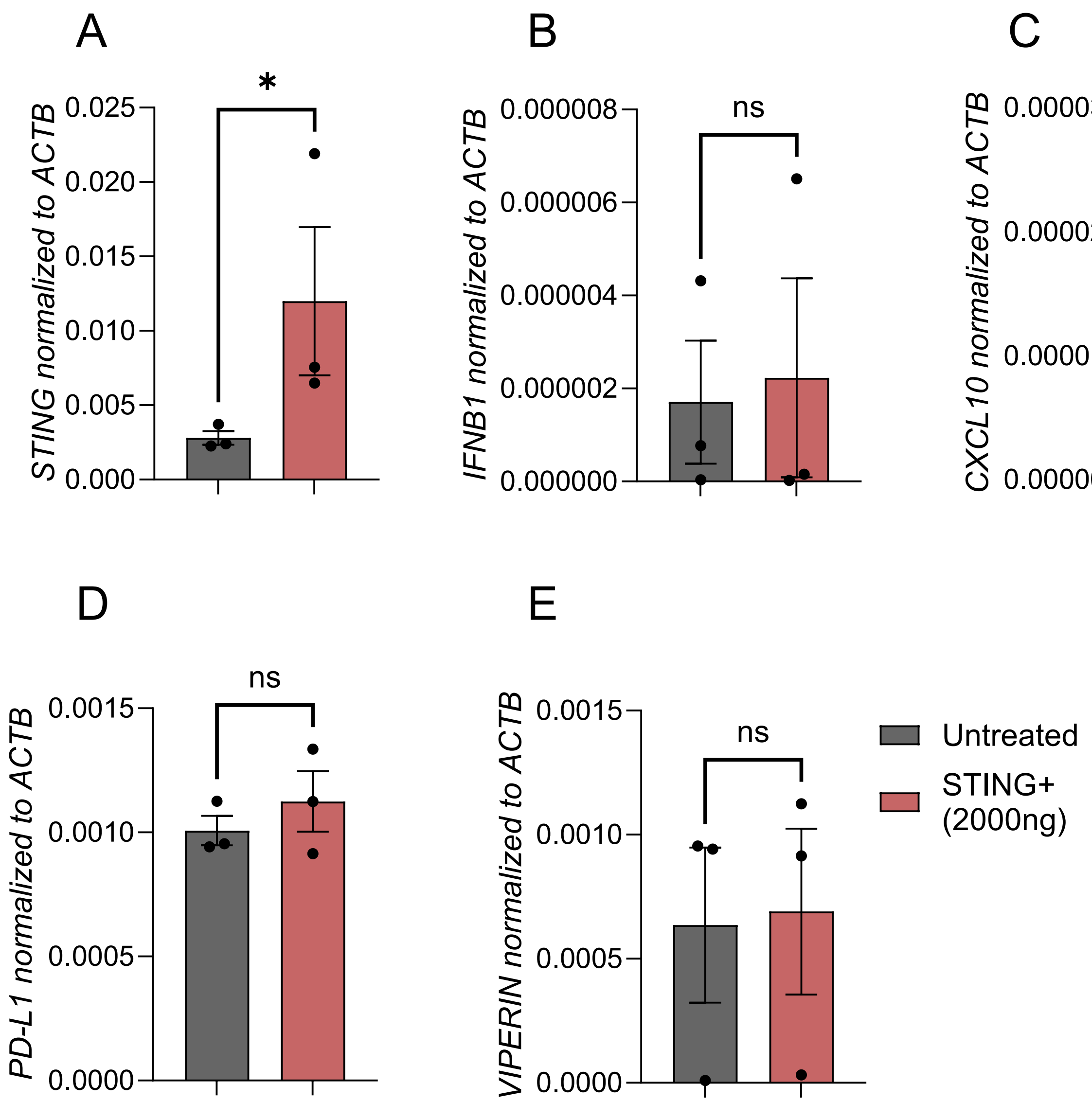

Supplementary Figure 8. Transcriptional activation of STING with LNPs upregulate downstream CXCL10

(**A-E**) CRISPRa of STING (STING+) with LNP delivery was performed in MFS1. mRNA was isolated at day three post CRISPRa and expression of indicated genes were measured by RT-qPCR using *ACTB* as a reference gene. A ratio paired t-test was performed. $p < 0.05$ *, $p < 0.01$ **, $p < 0.001$ ***, $p < 0.0001$ ****.

Figure S9. Godsk et al.

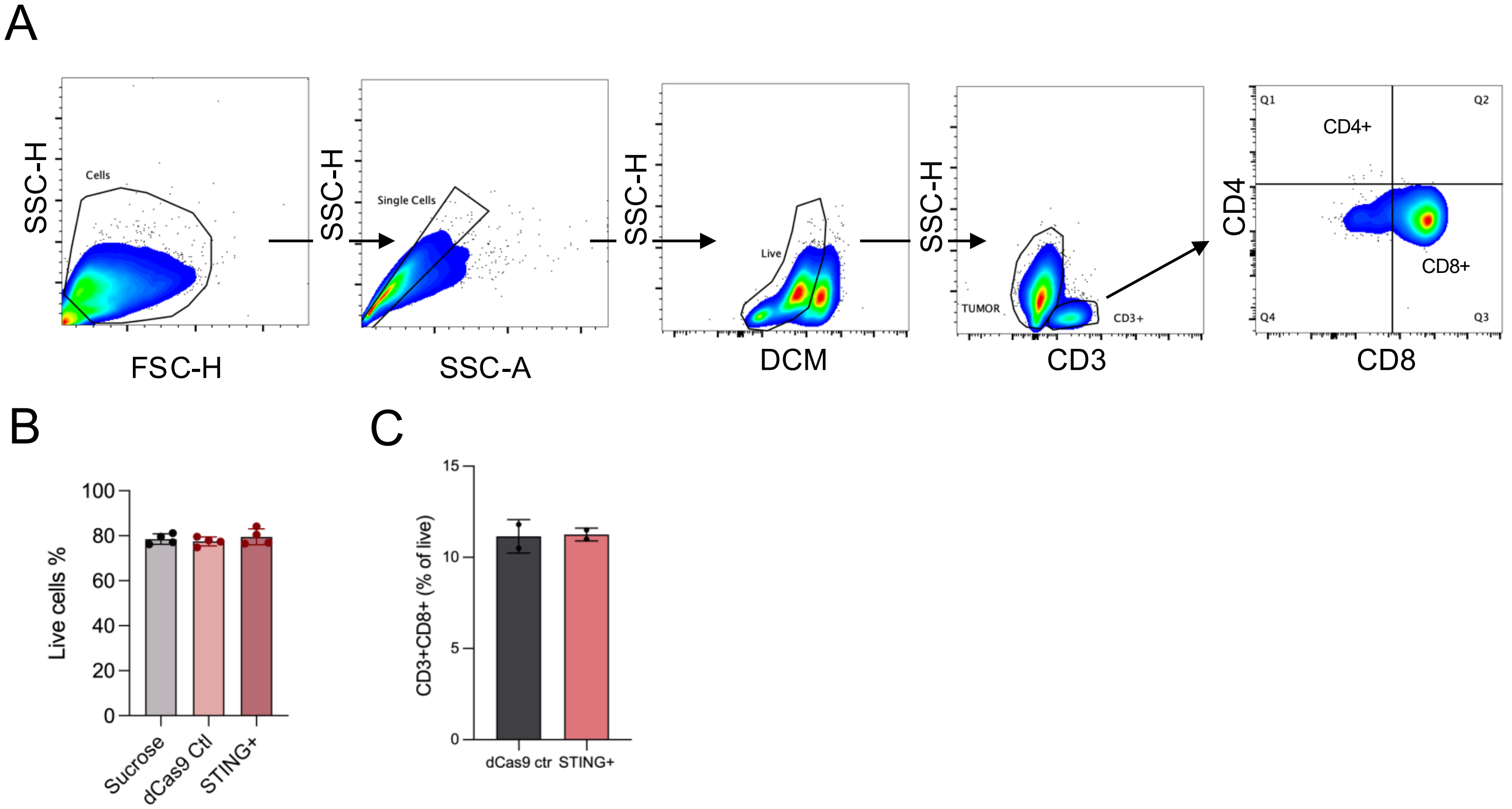

Supplementary Figure 9. Spheroid infiltration gating strategy.

(A) FSC-H and SSC-H were used to identify spheroid-derived single cells. Using SSC height versus area, the singlets were gated. The live cells were subsequently identified as dead cell marker (DCM)-negative cells. By using CD3, the infiltrating lymphocytes were identified from the tumor cells, forming the spheroid. The tumor infiltrating lymphocyte (TIL) population was further classified as CD4+ and CD8+. Note that most of our expanded TILs are CD8+. **(B)** Frequency of live cells in MFS1 patient-derived spheroids 3 days post LNP delivery. Sucrose consists of vehicle without added LNP while dCas9 Ctlr consists of LNPs formulated with dCas9 as control of LNPs baseline and inherent toxicity. **C)** Infiltration of CD3+CD8+ cells in spheroids with or without CRISPRa for STING after 48 hours of co-culture determined using flow cytometry.

Figure S10. Godsk et al.

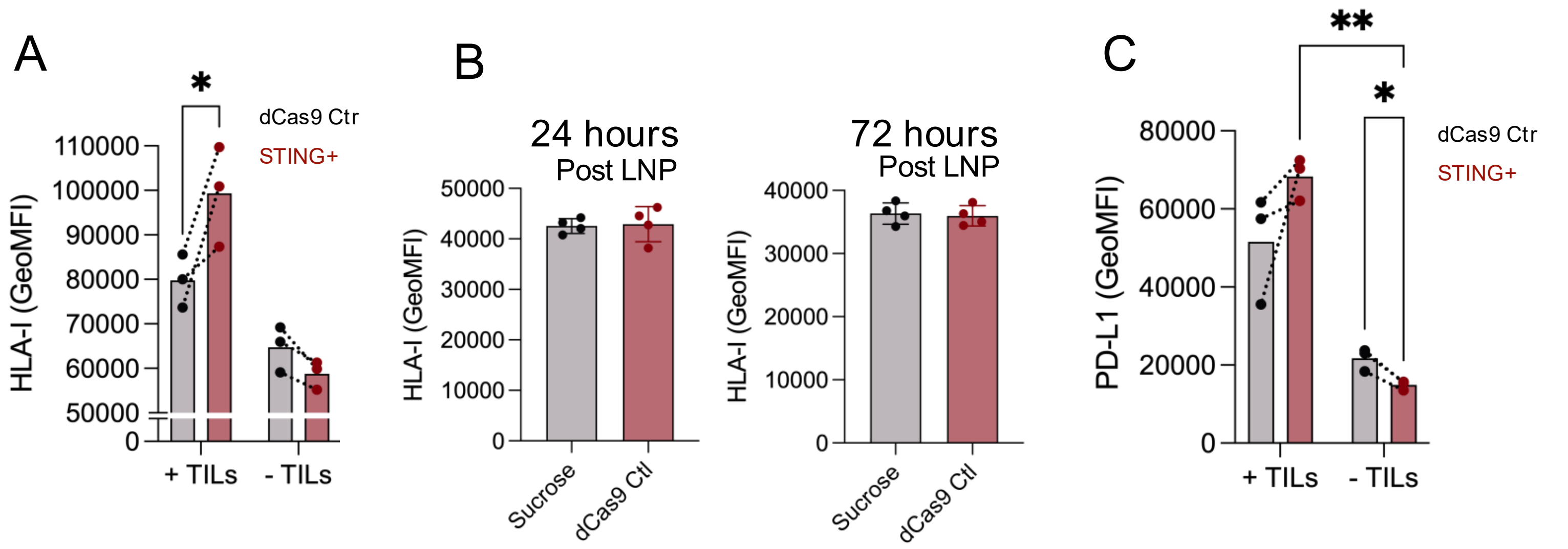

Supplementary Figure 10. HLA-I and PD-L1 expression in MFS1 spheroid patient cultures upon STING restoration and TIL co-culture. (A) Quantification of HLA-I expression in tumor cells from MFS1 spheroids co-cultured with autologous TILs (+TILs) or cultured without TILs (–TILs) over a 6-day period. (**B)** HLA-I expression in tumor cells from MFS1 patient-derived spheroids cultured for 24 h or 72 h under control conditions, including vehicle (sucrose) and dCas9 control LNPs lacking guide RNA. **(C)** PD-L1 expression in tumor cells from MFS1 spheroids co-cultured with autologous TILs (+TILs) or cultured without TILs (–TILs) over a 6-day period. C. Two-way ANOVA was used for A and C, while a Student t-test was employed for B. $p < 0.05$ *, $p < 0.01$ **, $p < 0.001$ ***, $p < 0.0001$ ****.
