# Supplementary material for: Restoring STING expression in soft tissue sarcoma increases activation and function of tumor-infiltrating lymphocytes
Source: Cancer Immunol Immunother. 2026 Jun 21;75(10):227. doi: 10.1007/s00262-026-04455-3 (PMC13578154; doi:10.1007/s00262-026-04455-3)
Supplement: Supplementary file 2 [file 262_2026_4455_MOESM2_ESM.docx]

*Supplementary Table 1: Genomic markers used for immune cell quantification*

| **Cell type** | | **Markers** | |
| --- | --- | --- | --- |
|  | B cells |  | *BLK, CD19, MS4A1, TNFRSF17, FCRL2, PNOC, SPIB, TCL1A* |
|  | CD45 cells |  | *PTPRC* |
|  | CD8 T cells |  | *CD8A, CD8B* |
|  | Cytotoxic cells |  | *CTSW, GNLY, GZMA, GZMB, GZMH, KLRB1, KLRD1, KLRK1, PRF1, NKG7* |
|  | Dendritic cells |  | *CCL13, CD209, HSD11B1* |
|  | Exhausted CD8 |  | *CD244, EOMES, LAG3, PTGER4* |
|  | Macrophages |  | *CD163, CD68, CD84, MS4A4A* |
|  | Mast cells* |  | *MS4A2, TPSAB1, CPA3, HDC, TPSB2* |
|  | Neutrophils* |  | *CSF3R, S100A12, CEACAM3, FCAR, FCGR3B, FPR1, SIGLEC5* |
|  | NK CD56dim cells* |  | *IL21R, KIR2DL3, KIR3DL1, KIR3DL2* |
|  | NK cells |  | *NCR1, XCL2, XCL1* |
|  | T cells |  | *CD3D, CD3E, CD3G, CD6, SH2D1A, TRAT1* |
|  | Th1 cells* |  | *TBX21* |
|  | Treg |  | *FOXP3* |
|  | CD4 cells |  | *IGFBP4, ITM2A, AMIGO2, TRAT1, CD40LG, ICOS, RCAN3* |

*Score used for calculating the Danaher score, but not included in the figures

*Supplementary Table 2: TCGA abbreviations for cancers*

| **Abbreviation** | | **Full name** | |
| --- | --- | --- | --- |
|  | BLCA |  | *Bladder urothelial carcinoma* |
|  | BRCA |  | *Breast invasive carcinoma* |
|  | CESC |  | *Cervical and endocervical cancer* |
|  | COAD |  | *Colon adenocarcinoma* |
|  | ESCA |  | *Esophageal carcinoma* |
|  | HNSC |  | *Head and neck squamous cell carcinoma* |
|  | KIRC |  | *Kidney clear cell carcinoma* |
|  | LUAD |  | *Lung adenocarcinoma* |
|  | READ |  | *Rectum adenocarcinoma* |
|  | SARC |  | *Sarcoma* |
|  | SKCM |  | *Skin cutaneous melanoma* |
|  | STAD |  | *Stomach adenocarcinoma* |
|  | UCEC |  | *Uterine corpus endometrioid carcinoma* |

*Supplementary Table 3: Primers used for RT-qPCR*

| **Target gene** | | **Primers** | |
| --- | --- | --- | --- |
|  | *STING1* |  | *Fw: CATGGGCTGGCATGGTCATA*  *Rev: AACCCGATCCTTGATGCCAG* |
|  | *cGAS* |  | *Fw: CTGCGCATTCAAAACTGGCT*  *Rev: TCGATGTGAGAGAAGGATAGCC* |
|  | *TBK1* |  | *Fw: GCAGATCAGGAAAAGTGTTGGG*  *Rev: AATGTTGTGCCAGCCTTCCA* |
|  | *IRF3* |  | *Fw: ATAAGCCAGACCTGCCAACC*  *Rev: GAGGTGTCTGGCTGGGAAAA* |
|  | *CXCL10* |  | *Fw: GAACCTCCAGTCTCAGCACC*  *Rev: GCAGGTACAGCGTACAGTTCT* |
|  | *IFNB1* |  | *Fw: TGCTCTCCTGTTGTGCTTCT*  *Rev: AGCCTCCCATTCAATTGCCA* |
|  | *PDL1* |  | *Fw: TGCCGACTACAAGCGAATTACTG*  *Rev: CTGCTTGTCCAGATGACTTCGG* |
|  | *VIPERIN* |  | *Fw: GCATCGTGAGCAATGGAAGC*  *Rev: CGTCATGTCCTCTTCCACGT* |
|  | *ACTB* |  | *Fw: GCACTCTTCCAGCCTTCCTT*  *Rev: CTCCTTCTGCATCCTGTCGG* |
|  | *EIF2B2* |  | *Fw: CAGAGAGGGCAGGAGGATGA*  *Rev: CCTGCTGATCACTCTCGTCG* |

*Supplementary Table 4: Guide RNAs used for CRISPR mediated transcriptional activation of STING*

| **Guide RNA** | | **Sequence** | |
| --- | --- | --- | --- |
|  | *sgRNA#1* |  | *GGUUCCUACCUCCCUUCCUG* |
|  | *sgRNA#2* |  | *UUUACUGGUGCUGGGAAGGA* |
|  | *sgRNA#3* |  | *GAGUGUGUGGAGUCCUGCUC* |
|  | *sgRNA#4* |  | *CAGAAACCGGCAGGCUCUCU* |
|  | *sgRNA#5* |  | *GAGGAGGGGCACAGAGGAAU* |

*Supplementary Table 5: Antibodies used for flow cytometry*

| **Specificity** | | **Fluorochrome conjugated** | | **Reference** | **Company** |
| --- | --- | --- | --- | --- | --- |
|  | STING |  | Alexa Fluor 647 | 564836 | BD |
|  | STING |  | BV421 | 564966 | BD |
|  | CD3 |  | Brilliant Violet 650 | 563852 | BD |
|  | CD4 |  | Super Bright 436 | 62-0049-42 | Invitrogen |
|  | CD8a |  | PE-Cy7 | 25-0088-42 | Invitrogen |
|  | PD-L1 |  | PE-Fire810 | 329755 | Biolegend |
|  | PD-L1 |  | BV785 | 329736 | Biolegend |
|  | HLA-ABC |  | PE | 311406 | Biolegend |
